# Analysis of the Secretome of Apoptotic Peripheral Blood Mononuclear Cells: Impact of Released Proteins and Exosomes for Tissue Regeneration

**DOI:** 10.1038/srep16662

**Published:** 2015-11-16

**Authors:** Lucian Beer, Matthias Zimmermann, Andreas Mitterbauer, Adolf Ellinger, Florian Gruber, Marie-Sophie Narzt, Maria Zellner, Mariann Gyöngyösi, Sibylle Madlener, Elisabeth Simader, Christian Gabriel, Michael Mildner, Hendrik Jan Ankersmit

**Affiliations:** 1 Department of Thoracic Surgery, Medical University of Vienna, Austria; 2 Christian Doppler Laboratory for Cardiac and Thoracic Diagnosis and Regeneration, Medical University of Vienna, Austria; 3 Department of Cell Biology and Ultrastructure Research, Center for Anatomy and Cell Biology, Medical University of Vienna, Vienna, Austria; 4 Department of Dermatology, Research Division of Biology and Pathobiology of the Skin, Medical University of Vienna, Austria; 5 Christian Doppler Laboratory for Biotechnology of Skin Aging, Medical, Austria; 6 Centre of Physiology and Pharmacology, Institute of Physiology, Medical University of Vienna, Austria; 7 Department of Cardiology, Medical University of Vienna, Vienna, Austria; 8 Molecular Neuro-Oncology Research Unit, Department of Pediatrics and Adolescent Medicine and Institute of Neurology, Medical University of Vienna, Austria; 9 Red Cross Blood Transfusion Service of Upper Austria, Linz, Austria

## Abstract

We previously showed that, when peripheral blood mononuclear cells (PBMCs) were stressed with ionizing radiation, they released paracrine factors that showed regenerative capacity *in vitro* and *in vivo*. This study aimed to characterize the secretome of PBMCs and to investigate its biologically active components *in vitro* and *vivo*. Bioinformatics analysis revealed that irradiated PBMCs differentially expressed genes that encoded secreted proteins. These genes were primarily involved in (a) pro-angiogenic and regenerative pathways and (b) the generation of oxidized phospholipids with known pro-angiogenic and inflammation-modulating properties. Subsequently, *in vitro* assays showed that the exosome and protein fractions of irradiated and non-irradiated PBMC secretome were the major biological components that enhanced cell mobility; conversely, secreted lipids and microparticles had no effects. We tested a viral-cleared PBMC secretome, prepared according to good manufacturing practice (GMP), in a porcine model of closed chest, acute myocardial infarction. We found that the potency for preventing ventricular remodeling was similar with the GMP-compliant and experimentally-prepared PBMC secretomes. Our results indicate that irradiation modulates the release of proteins, lipid-mediators and extracellular vesicles from human PBMCs. In addition our findings implicate the use of secretome fractions as valuable material for the development of cell-free therapies in regenerative medicine.

Regenerative medicine that aims to restore damaged or dysfunctional tissue has emerged as a new branch of research in the last century worldwide^1^. Despite major advances in drug therapies, surgical interventions, and organ transplantation, tremendous problems remain unresolved for the regeneration of injured organs, including the myocardium, kidney, the central nervous system, lung, and skin^2^. The use of stem cells as therapeutic agents has yielded promising results in preclinical and clinical studies in several experimental settings. However, the mode of action underlying stem cell transplantation continues to be debated. In recent years, it has become commonly accepted that transplanted stem cells release paracrine factors that enhance the capacity for endogenous regeneration, rather than directly replacing injured cells^3^,^4^. Therefore, the use of paracrine factors instead of administering living, proliferating, potentially pluripotent stem cell populations would represent a great advantage with respect to meeting regulatory restrictions and safety issues.

Although the majority of cell therapy studies were performed with stem cells from different origins, we and others have shown that stressed peripheral blood mononuclear cells (PBMCs) could also promote tissue protection and repair through paracrine activities^5^,^6^,^7^,^8^,^9^,^10^,^11^. The secretome of stressed PBMCs has been shown to enhance angiogenesis and wound healing *in vitro* and *in vivo*^10^. These activities promoted regeneration of the myocardium^5^,^6^,^7^ and brain^12^ after acute ischemic injuries. In contrast to stem cells, which are available in limited cell numbers, large numbers of PBMCs can be readily obtained. Although several studies have demonstrated that the PBMC-derived secretome has biological effects on cardioprotection, angiogenesis, and wound healing, only a few studies have identified the paracrine factors involved^5^,^6^,^11^.

To gain a better understanding of the *in vitro* and *in vivo* effects of the PBMC secretome, it is necessary to analyze in detail the biological components present in conditioned medium (CM). The secretome of cultured PBMCs comprises proteins, lipids, and extracellular vesicles; thus, a multidimensional methodical approach must be implemented for this type of analysis. To date, several secreted proteins have been identified that exert cytoprotective and regenerative capacities^13^,^14^; thus, those proteins are thought to be important mediators in paracrine signaling. In addition, the lipids released in cell cultures have been shown to modulate immune function^15^, induce angiogenesis, and enhance wound healing by upregulating pro-angiogenic proteins (reviewed in^16^). More recently, extracellular vesicles, including microparticles and exosomes, have come into focus in regenerative medicine, because extracellular vesicles isolated from donor cells could interact with recipient cells, and they displayed pleiotropic immunological functions^17^. Recent studies have revealed that, when exosomes released from mesenchymal stromal cells were administered in injured animals, they induced neurogenesis following a stroke^18^, they induced cardioprotection after acute myocardial infarction, and they augmented angiogenesis and wound healing in a rodent skin burn model^19^. Extracellular vesicles mediate intercellular communication by delivering mRNAs, microRNAs (miRNAs), proteins, and lipids from one cell to another^20^,^21^. Furthermore, several reports showed that cell stressors, like hypoxia, could enhance the release of pro-angiogenic exosomes and augment their biological efficacy^22^,^23^.

In the present study, we aimed to characterize in detail the secretome of non-irradiated and irradiated PBMCs with a combination of methods, including transcriptomics, lipidomics, and functional *in vitro* assays. Furthermore, we evaluated whether a viral-cleared, PBMC secretome, prepared in compliance with good manufacturing practice (GMP) guidelines, retained its preventative potency in a porcine, closed-chest-reperfusion, acute myocardial infarction (AMI) model.

We demonstrated that irradiation induced the expression of pro-angiogenic factors, the shedding of microparticles and exosomes, and the production and release of oxidized phospholipids, either in solution or incorporated into extracellular vesicles. We showed *in vitro* that exosomes and proteins were the two major biologically active components present in the secretome of irradiation-induced PBMCs. These components enhanced fibroblast and keratinocyte cell migration and the release of pro-angiogenic factors that are considered hallmarks of tissue regeneration. Finally, we demonstrated *in vivo* that “cell free” regenerative medicine that met the requirements of regulatory authorities showed potency in preventing ventricular remodeling after an experimental AMI.

## Materials and Methods

### Ethics statement

This study was performed in accordance with the Ethics Committee of the Medical University of Vienna (EK: 1236;2013) and according to the principles of the Helsinki Declaration and Good Clinical Practice. Written, informed consent was obtained from all participants. All experimental protocols were approved by the Ethics Committee of the Medical University of Vienna (EK: 1236;2013).

### Cell separation and irradiation

Human peripheral blood mononuclear cells (PBMC) were isolated from four healthy male volunteers by venous blood draw and density gradient centrifugation with Ficoll-Paque (GE Healthcare Bio-Sciences AB, Sweden). PBMCs (25 × 10^6^ cells/ml) were resuspended in serum-free medium (CellGro, CellGenix, Freiburg, Germany). An automated cell counter (Sysmex Inc., USA) was used to determine cell count. PBMCs were gamma-irradiated with 60 Gy to induce apoptosis. Induction of apoptosis was confirmed by annexin V-fluorescein/propidium iodide (FITC/PI) co-staining (Becton Dickinson, Franklin Lakes, NJ, USA) using a flow cytometer. At 20h after irradiation 58% of PBMCs were annexin V/PI positive (supplementary Fig. S1). CM was collected from cultures at 2, 4, and 20 h time points, and then, centrifuged (500 × *g* for 9 min) to remove cell debris. CM was stored at −80 °C for subsequent protein and lipid analyses. Fresh CM was used for microparticle and exosome separations.

### RNA isolation

PBMCs were collected immediately after isolation (0 h) and after culturing for the indicated times (2, 4, and 20 h) after treatment (radiation or no radiation). From these samples (25 × 10^6^ cells/sample), total RNA was isolated with Trizol^®^ Reagent (Invitrogen, Carlsbad, CA). RNA was quantified with a NanoDrop 1000 spectrophotometer (Peglab, Erlangen, Germany). RNA quality was verified with an Agilent 2100 Bioanalyzer (Agilent, Böblingen, Germany). All RNA samples had an integrity score between 5.7 and 10. Overall, 28 samples were generated from the four individual donors.

### Microarray analysis

Microarray expression profiling was performed with an Agilent, whole human genome oligo microarray, 8×60 Kb (G4851A; #028004; Agilent Technologies), which contained 27,958 target genes (Entrez IDs) and 7,419 lincRNAs. Staining and scanning were performed according the Agilent expression protocol (Agilent Technologies). Microarray gene analysis was performed by Miltenyi (Miltenyi Biotec, GmBH, Germany), according to the MIAME guidelines^24^.

Background-corrected fluorescence intensity values of microarray data were statistically analyzed with Genespring v.11.5 software. Expression values were log_2_-transformed and normalized with the quantile-normalization method. Transcripts were further processed only when at all time points, the expression in at least one of the two conditions (irradiated or non-irradiated) was above the 40^th^ percentile of the average expression measured over all samples. The threshold of 40% was chosen, because approximately 30–60% of all human genes are expressed^25^. A paired student’s t-test, in combination with a false discovery rate <5%, were used to calculate significant differences. Only genes that displayed a >2-fold change (FC) in expression were used for functional analyses. Array data was submitted to GEO (http://www.ncbi.nlm.nih.gov/geo/) under accession number GEO: GSE55955.

### Secreted factor prediction

**Figure 1:**
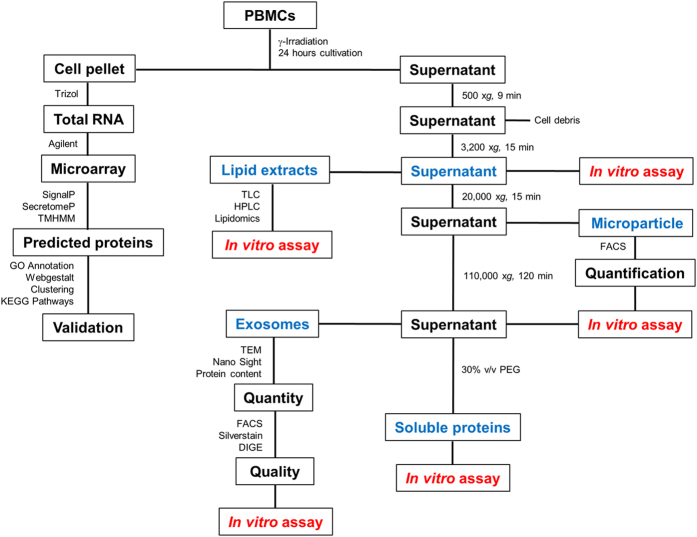
Schematic overview of experimental workflow. PBMCs were either gamma-irradiated with 60 Gy or not irradiated. After culturing for the indicated times, cells were centrifuged to separate the cell pellet and CM supernatant. The pellet was used to extract cellular RNA, which was used for microarray analysis. The CM supernatant was processed to separate and isolate different molecular components. The steps highlighted in blue indicate samples used for *in vitro* assays. The different methods and bioinformatics tools used for sample analyses are indicated at the appropriate links.

To identify transcripts that encoded secreted proteins, we used three web-based programs: SecretomeP 2.0, SignalP 4.1, and TMHMM 2.0. The workflow of data analysis is shown in Fig. 1. The SignalP program predicts the presence and location of signal peptide cleavage sites in amino acid sequences 26. Based on this information, a specific threshold (D-cutoff score ≥0.45) is generated, which predicts secretory proteins. Currently, the SignalP program shows the best performance and accuracy compared to similar available algorithms^27^. SecretomeP predicts whether a protein is secreted via a non-classical pathway, based on post-translational and localization information obtained from different protein-prediction servers. The information on protein characteristics is expressed with a neural network score (NN-score), and proteins with a NN-score ≥0.5 (cut-off value) are considered to be secreted via a non-classical pathway. TMHMM 2.0 predicts transmembrane helices in proteins, based on a hidden Markov model. This method discriminates between soluble and membrane proteins with a high degree of accuracy^28^.

We used these three different programs to predict transcripts that encoded secretory proteins. We analyzed transcripts that were upregulated in at least 2 of 3 time points compared to control conditions (0 h), in irradiated or non-irradiated PBMCs.

### Functional annotation clustering and pathway analysis

The identified transcripts that encoded secreted proteins were classified according to a web-based Gene Toolkit (WEBGESTAL) (http://bioinfo.vanderbilt.edu/webgestalt/analysis.php). The toolkit employed gene ontology term enrichment (Go-term) and the Kyoto Encyclopedia of Genes and Genomes (KEGG) to determine functional pathways^29^. PANTHER (Protein Analysis Through Evolutionary Relationships; http://www.pantherd.org) was used for phylogenetic inferences.

### Visualization of protein-protein interactions

To visualize known and predicted protein-protein interactions, we used the web-based database, STRING v9.1 (Search Tool for the Retrieval of Interacting Genes/Proteins).

### Exosome enrichment

Exosomes were purified from the CM of irradiated and non-irradiated human PBMCs (25 × 10^6^ cells) after culturing for 20 h in serum-free cell culture media. We used either a total exosome isolation kit (Invitrogen), according to the manufacturer’s instructions, or an ultracentrifuge centrifugation protocol. In both cases, CM was centrifuged at 500 × *g* for 2 min to remove cells, followed by centrifugation at 3500 × *g* for 15 min to eliminate debris, and then, at 20,000 × *g* for 15 min to eliminate microparticles. Next, the CM was filtered through 0.2-μm pore filters. For exosome isolation with the Invitrogen isolation kit, we transferred 1 ml CM into a new tube and added 0.5 ml total exosome reagent. After rigorous vortexing, the solution was incubated at 4 °C overnight, followed by centrifugation at 10,000 × *g* for 1 h at 4 °C. For exosome isolation with the ultracentrifuge protocol, 10 ml of microparticle-depleted CM was centrifuged for 120 min at 110,000 × *g* in a SW 41 swinging bucket ultracentrifuge (Beckman Coulter, Brea, California, USA). The pelleted exosomes were eluted in 500 μl PBS. The absolute number of exosomes was assessed with a NanoSight, NS500 instrument. All centrifugation procedures were performed at 4 °C.

### Exosome flow cytometry

Exosomes freshly isolated from cell culture media were labeled with a commercially available, human CD63 isolation/detection kit (Invitrogen), according to the manufacturer’s instructions. Briefly, 50 μl of pre-enriched exosomes were mixed with dynabeads coated with anti-CD63 antibody. The mixture was incubated overnight under gentle agitation at 4 °C. After several washing steps, exosomes bound to anti-CD63 beads were resuspended in 300 μl PBS with 0.1% BSA. Then, 100 μl of bead-bound exosomes were incubated with 4 μl anti-CD63-FITC and anti-CD9-PE or matching isotype controls (BioLegends). After 45 min, the labeled exosomes were washed twice and resuspended in 500 μl PBS with 0.1% BSA. Then, exosomes were detected on a FACSAria flow cytometer (Becton Dickinson). Data were analyzed with FlowJo Software (Tree Star, Inc, Ashland, OR, USA).

### Microparticle preparation

Microparticles were isolated from 1 ml CM from irradiated and non-irradiated human PBMCs (25 × 10^6^ cells). The CM was initially centrifuged at 500 × *g* for 2 min to separate the pellet from the supernatant. The supernatant was spun at 3500 × *g* for 15 min to eliminate debris. The resulting cell-free CM was stored at −20°C.

### Microparticle flow cytometry

Microparticles in CM samples were analyzed with a FACSAria flow cytometer. Briefly, 250 μl cell-free CM was centrifuged at 20,000 × *g* for 15 min at 4 °C to pellet the microparticles. Then, 225 μl supernatant was removed, and the microparticle pellet was resuspended in 200 μl of filtered annexin binding buffer. The annexin binding buffer had been previously filtered through 0.2-μm pore filters to remove background noise.

Microparticles are lipid vesicles shed from the cell membrane. We employed fluorescently-labeled annexin V to label microparticles, because annexin V spontaneously binds to cell membranes in the presence of calcium. Annexin V conjugated to FITC (4 μl, eBioscience) was resuspended in 100 μl annexin binding buffer and centrifuged at 20,000 × *g* for 15 min. Then, 80 μl were added to 100 μl resuspended microparticles, and incubated for 20 min at room temperature. Then, the annexin-bound were pelleted at 20,000 × *g* for 15 min, Next, the supernatant (180 μl) was discarded, and the microparticle pellet was resuspended in 480 μl annexin binding buffer. In summary, the microparticles present in 100 μl CM were diluted in 500 μl buffer for FACS analysis.

We used Megamix-Plus SSC beads (BioCytex, Marseille, France) to determine the gating for microparticles. The forward scatter (FSC), side scatter (SSC), and FITC FL-1 were set to log mode. Briefly, beads with 0.16 μm, 0.2 μm, 0.24 μm, and 0.5 μm diameters were detected on an SSC/FL1 plot. We next selected bead regions, and back-gated them onto the FSC/SSC plot. Then, the microparticle gate was set based on the FSC/SSC plot. A small microparticle gate was applied by using a particle size of 0.2 μm–0.3 μm (small microparticles) and a large microparticle gate was set at a particle size of 0.3 μm–0.5 μm (large microparticles). Particles that were 0.16 μm or smaller were not counted to remove false-positive events based on inaccurate measurements.

Nonspecific annexin V labeling was evaluated by preparing microparticles in PBS without calcium, which yielded no annexin V-positive events. As a negative control, CM was filtered through a 0.2 μm filter, which removed >99.9% of the particles that might pass through the small microparticle gate.

During the entire analysis, the lowest available flow rate was chosen. TrueCount tubes (BD Biosciences) were used for detection at SSC/FL1 to measure the absolute counts of microparticles.

Absolute numbers of microparticles were calculated as recommended in the manufactures instructions. We acquired 100,000 events, and the data were analyzed with FlowJo Software (Tree Star, Inc, Ashland, OR, USA).

### Silver staining

Exosomes were isolated with an exosome isolation kit. Briefly, exosomes were isolated from 0.5 ml CM by binding to anti-CD63 attached to magnetic beads. Exosomes were suspended in an SDS-PAGE loading buffer, lysed by sonication, and centrifuged. The anti-CD63 beads were removed with a magnet. Lysates with 20 μg protein content were separated by PAGE and visualized with silver stain.

### Lipid extraction and thin layer chromatography

Total lipid extracts were subjected to thin-layer chromatography (TLC) analyses on Silica gel 60 TLC plates (Merck, Vienna, Austria). Briefly, 25 × 10^6^ cells were cultured for 20 h after irradiation. CM was isolated, lipids were extracted, and 1/10 of the total extract from one ml CM was spotted onto the TCL plate.

For TLC separation of total lipids, we used the method described by Pappinen . As the lipids migrated through the matrix, the following solvent systems were used sequentially: chloroform/methanol/water 40:10:1 (v/v/v) to 10 cm; chloroform/methanol/acetic acid 190:9:1 (v/v/v) to 16 cm; and hexane/diethylether/acetic acid 70:30:1 (v/v/v) to 20 cm.

In all experiments, the plates were dried under air stream before they were developed with a new mobile phase. Lipids were visualized by exposing the plates to 10% copper sulfate in an 8.5% aqueous solution of ortho-phosphoric acid, and subsequently, drying and heating at 150 °C. Lipid classes were identified by comparing the bands of skin-equivalent lipids with standards for triglycerides, free fatty acids, ceramides, sodium cholesterylsulphate, sphingomyelin (all Sigma), and phosphatidylcholines (PCs; Avanti Lipids, Alabaster, AL). ImageJ 1.45 software (National Institutes of Health, Bethesda, MD, USA) was used for semi-quantitative analyses of lipids. The mean pixel intensities of lipids from the CM of irradiated PBMCs was plotted against those from the CM of non-irradiated PBMCs.

For mass spectrometry analyses, lipids were extracted with a liquid-liquid extraction procedure, as described recently^30^. Briefly, lipid extracts from complete cell culture supernatants were spiked with 1,2-dinonanoyl-sn-glycero-3-phosphocholine (DNPC; Avanti Lipids, Alabaster, Alabama) for standardization. Then, neutral lipids and fatty acids were removed with three rounds of hexane extraction. Reverse-phase chromatography, and subsequently, an online electronspray ionization-tandem mass spectrometry procedure were used to analyze non-oxidized and oxidized phosphocholines (oxPCs), with *m/z* 184 as a diagnostic fragment marker for PC^30^. Sample peak areas were normalized to the peak areas of the internal standard, DNPC.

### Protein analysis

Irradiated and non-irradiated PBMCs (25 × 10^6^/ml each) were cultured for 20 h. The CM was collected and analyzed with commercially available, enzyme-linked immunosorbent assay (ELISA) kits. We quantified the following proteins: angiogenin, CXCL13, PDGF-AA, PDGF-BB (Duoset, R&D Systems, Minneapolis, USA), complement C3, thrombospondin-1, neuropilin, and adrenomedullin (BG Bluegene Biotech). We used various biological components of the CM to stimulate fibroblasts (FBs) and keratinocytes (KCs), and then quantified the secretion of cytokines, CXCL1 and CXCL8 (Duoset, R&D Systems).

### Transmission electron microscopy

Irradiated and non-irradiated PBMCs were cultured for 20 h. Then, PBMCs were examined with transmission electron microscopy (TEM), performed as described previously^31^.

### Isolation of biological components present in conditioned media

For *in vitro* assays, the CM samples from PBMCs of four donors were pooled before isolating biological components. In total, two independent experiments were performed by drawing blood samples from eight donors donor, and producing two independent CM pools. Microparticles and exosomes were purified as described. Lipids and proteins were isolated from the CM after removing the exosomes; the exosomes also contained lipid and protein fractions that were not analyzed here. Thus, the lipid and proteins analyzed were either soluble or attached to vesicles other than exosomes. Lipids were isolated as described above and in a previous study^32^. Proteins were precipitated with 30% v/v polyethylene glycol. The precipitated proteins were centrifuged for 15 min at 20,000 × *g*. Aliquots were stored at −80 °C before experiments were performed.

### Generation of pathogen-reduced cell culture supernatant

Pathogens were reduced in CM, as described previously^12^.

### Large animal *in vivo* experiments

Large animal studies were performed as described previously^6^. Animal investigations were carried out in accordance with the “Position of the American Heart Association on Research Animal Use”, as adopted by the AHA on November 11, 1984. The study was approved by the Ethics Committee on Animal Experimentation at the University of Kaposvar, Hungary.

### Cell culture and *in vitro* stimulation assay

Human dermal FBs (Lonza) were cultured in DMEM (Gibco, BRL, Gaithersburg USA), supplemented with 10% fetal bovine serum, 25 mM L-glutamine (Gibco), and 1% penicillin/streptomycin (Gibco). Human primary KCs were cultured in KC-growth medium (KGM, Lonza). For stimulation assays, 3 × 10^5^ FBs or KCs were seeded in 12-well plates. After reaching 80% confluence, cells were washed once with PBS. Unprocessed CM, microparticles, exosomes, proteins, lipids or CM depleted for microparticles and exosomes were harvested from (1) control medium cultivated without cells, (2) CM from 2.5 * 10^6^ non-irradiated PBMCs, or (3) CM from 2.5 * 10^6^ irradiated PBMCs. To obtain microparticle and exosome free CM, CM was centrifuged at 500 × g for 2 min, at 3500 × g for 15 min and at 110,000 × *g* for 120 min. All single components were resuspended in either DMEM or KGM basal medium. Each biological component was then added to FB and KC cultures. After 6 h, cells were washed once with PBS, and RNA was isolated with the RNAeasy kit (Qiagen, Hilden, Germany) according to the manufacturer’s instructions. Cell stimulation assays were performed twice, with two different preparations of stimulating components.

### *In vitro* scratch assay

FBs and KCs (3 × 10^5^ each) were seeded in 6-well plates. After reaching 100% confluence, cells were scratched horizontally and vertically with a pipette-tip. Then, cells were washed once with PBS. The scratches were investigated under the microscope, and four areas were marked for photographs. The first photographs of those 4 areas were acquired immediately (initial wound size), and the clear areas were marked. Unprocessed CM, microparticles, exosomes, proteins, or lipids were resolved from (1) control medium cultivated without cells, (2) CM from non-irradiated PBMCs, and (3) CM from irradiated PBMCs, and resuspended in either DMEM or KGM. Each biological component was then added to the scratch-wounds. Each component was diluted to achieve the content equivalent to that derived from 2.5 × 10^6 ^PBMCs/ml. The same areas of the scratch wounds were photographed again after 24 h (for FBs and KCs) and after 48 h (only for FBs). The wound closures were measured with ImageJ 1.45 software (National Institutes of Health, Bethesda, MD, USA). These wound repair assays were performed twice, with two different preparations of stimulating components.

### Quantitative reverse-transcriptase PCR (qPCR) analysis of mRNA

Total RNA was reverse-transcribed with the IScript cDNA synthesis kit (BioRad, Hercules, CA, USA), as indicated in the instruction manual. Then, qPCR was performed with the Light Cycler Master SYBR Green I kit (Roche Applied Science, Penzberg, Germany) on a Light Cycler 480 thermocycler (Roche Applied Science) as described previously^33^. The primer pairs were synthesized by Microsynth AG (Vienna, Austria; sequences in supplementary Table S1). The reference gene was beta-2-microglobulin (B2M).

### Statistical analysis

Data distributions were tested with the Kolmogorow-Smirnow-Test. An ANOVA with Bonferroni post hoc test for normally distributed data or a Kruskal-Wallis test with Dunns post hoc test was used to analyze results. A P-value < 0.05 was taken to indicate a significant difference (*p < 0.05; **p < 0.01; ***p < 0.001).

## Results

### Bioinformatics analysis of the secretome

Transcriptomic profiling and bioinformatics tools were used to identify proteins secreted from non-irradiated and irradiated PBMCs from four donors (Fig. 1). PBMCs were cultured for 2, 4, and 20 h; then, gene expression was profiled at each time point on DNA microarrays that covered 47,000 transcripts. Actively secreted factors were identified with the bioinformatics programs, SecretomeP, SignalP, and TMHMM.

**Figure 2:**
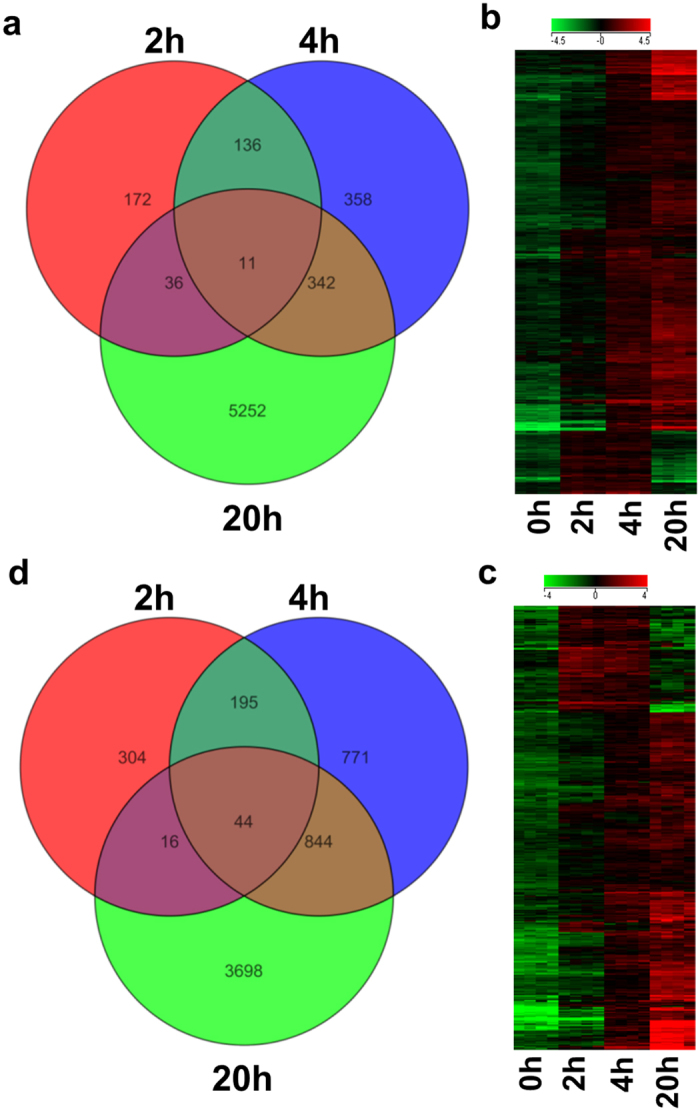
Analysis of changes in PBMC transcriptome. PBMCs were either irradiated or not irradiated *ex vivo*, before culturing for 2, 4, or 20 h. At the indicated time points, total RNA was isolated, and microarray analyses were performed to evaluate gene expression. (*Left*) Venn diagrams show the numbers of upregulated transcripts with expression changes >2-fold above expression after cell separation for (**a**) non-irradiated and (**c**) irradiated PBMCs. Each circle depicts the genes detected at the indicated time point; overlapping sections indicate the number of genes that were upregulated at multiple time points. (*Right*) The heatmaps show that a significant number of genes were upregulated in (**b**) non-irradiated and (**d**) irradiated PBMCs with a strong time dependence, and expression was most prominent at 20 h after cultivation; green = downregulated; red = upregulated in cultured samples; n = 4 for each time point.

We first identified transcripts that were upregulated during the cultivation period in either non-irradiated (Fig. 2a) or irradiated PBMCs (Fig. 2c). We only analyzed transcripts with a FC ≥ 2 compared to baseline values. The heatmaps displayed 525 and 1099 genes that were upregulated in at least two of the three time points in non-irradiated (Fig. 2b) and irradiated PBMCs (Fig. 2d), respectively. The bioinformatics analysis identified 167 transcripts that encoded actively secreted proteins in non-irradiated PBMCs and 213 that encoded secreted proteins in irradiated PBMCs (supplementary Table S1).

We then investigated the possible biological functions of these proteins with GO-term and KEGG pathway analyses. The 213 genes from the irradiated cells showed significant enrichment in genes involved in the biological processes of angiogenesis, wound healing, and leucocyte trafficking regulation (p < 0.05, supplementary Table S2). The 167 genes from the non-irradiated cells showed enrichment in genes involved in amino acid transport and endocrine regulation. These data suggested that gene expression shifted from metabolic processes in the non-irradiated state, towards tissue regeneration after irradiation.

### Confirmation of microarray data by qPCR and ELISA

A selected set of genes that encoded pro-angiogenic proteins that were upregulated in non-irradiated or irradiated PBMCs were validated with qPCR (supplementary Fig. S2A-S). We observed a time-dependent increase in gene expression; the highest expression was observed at 20 h after irradiation. We used ELISA to quantify protein content in the supernatant (supplementary Fig. S3A–H). Compared to non-irradiated PBMCs, the supernatants of irradiated PBMCs contained significantly higher concentrations of neuropilin, thrombospondin, CXCL13, and angiogenin, but similar concentrations of PDGF-AB, PDGF-BB, C3, and adrenomedullin.

### Lipid analysis

We investigated whether irradiation modulated the concentrations and composition of different CM lipid classes with TLC assays. Irradiated and non-irradiated PBMCs were cultured for 20 h, and CMs were analyzed. The TLC protocol^34^ was designed to quantify a broad range of different lipid classes. A representative TLC image is shown in supplementary Fig. S4A. Cell culture medium alone (no cells) showed no detectable lipids (line 3, marked “M”). Quantification of the TLC results showed that the CM of irradiated PBMCs contained significantly higher concentrations of phospholipids, cholesterol sulfate, cholesterol, free fatty acids, cholesterol esters, and triglycerides compared to CM from non-irradiated PBMCs (supplementary Fig. S4B).

### Ionizing radiation induces phospholipid oxidation

**Figure 3:**
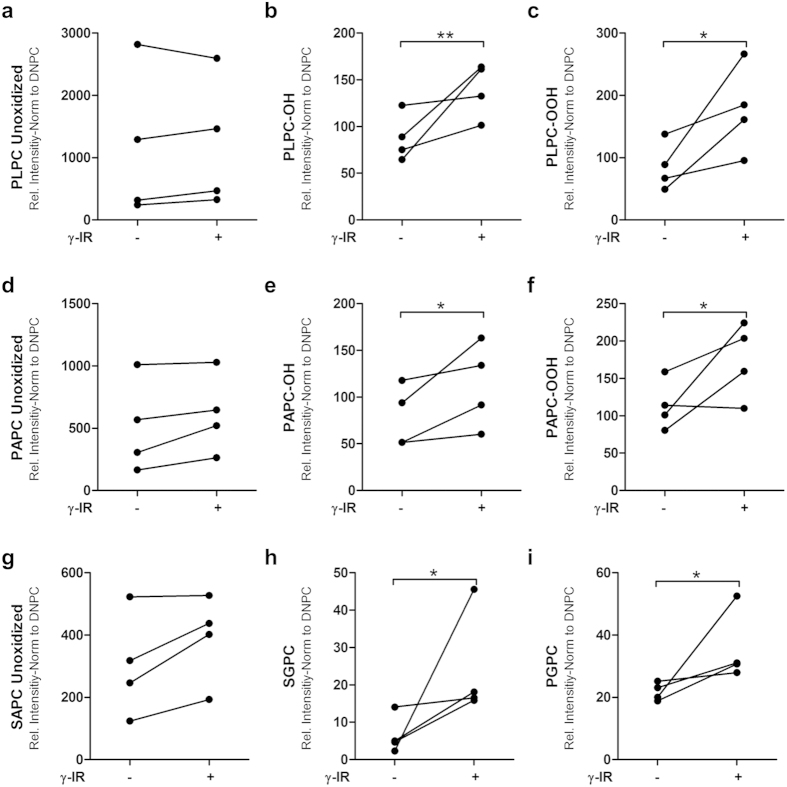
Ionizing radiation induces the release of oxidized phosphatidylcholines in human PBMCs. Irradiated (+) and non-irradiated (−) PBMCs were cultured for 24 h. Then, cells and cell debris were removed with serial centrifugations, and the lipids were separated with a chloroform/methanol extraction protocol. Lipids were analyzed with high pressure lipid chromatography to determine the presence of oxidation products. (*Left column*, a, d, g) The non-oxidized precursor phospholipids were comparable between irradiated and non-irradiated samples. (*Middle and right columns*, b, c, e, f, h, g, i) Oxidized lipid products were detectable in significantly higher concentrations in the CM of irradiated samples compared to non-irradiated samples. Data are expressed as the mean ± SD intensity, relative to that of the DNPC standard; Dots on each line display values of a single donor; n = 4. *p < 0.05; **p < 0.01

Previous studies have shown that UV radiation induced significant oxidation of PCs^35^. Here, we quantified oxPCs in the CM of irradiated and non-irradiated PBMCs at 20 h after irradiation. High pressure lipid chromatography-tandem mass spectrometry was used to detect selected oxidized phospholipid products that originated from selected, abundant PCs with polyunsaturated fatty acids in the sn-2 position and palmitic or stearic acid in the *sn*-1 position. Peak intensities were normalized to the level of DNPC, which served as an internal standard. The abundance of non-oxidized precursors, 1-palmitoyl-2-linoleoyl-sn-glycero-3-PC (PLPC), 1-palmitoyl-2-arachidonyl-PC (PAPC), and 1-stearoyl-2-arachidonoyl-sn-glycero-PC (SAPC), were not significantly different between the irradiated and non-irradiated samples (Fig. 3a,d,g, respectively). However, products with intact, but oxidized sn-2 chains, like PLPC-OH (Fig. 3b) and PLPC-OOH (Fig. 3c), were significantly more abundant after irradiation. We also observed a comparable significant increase in the oxidation of PAPC with irradiation (Fig. 3d–f). In addition, in the CM of irradiated PBMCs, we observed higher concentrations of oxidized lipids with fragmented chains, like 1-stearoyl-2-glutaroyl- sn -glycero-3-PC (SGPC), and 1-palmitoyl-2-glutaroyl- sn-glycero-3-PC (PGPC) compared to the CM of non-irradiated PBMCs (Fig. 3g–i).

### Ionizing radiation induces microparticle release

**Figure 4:**
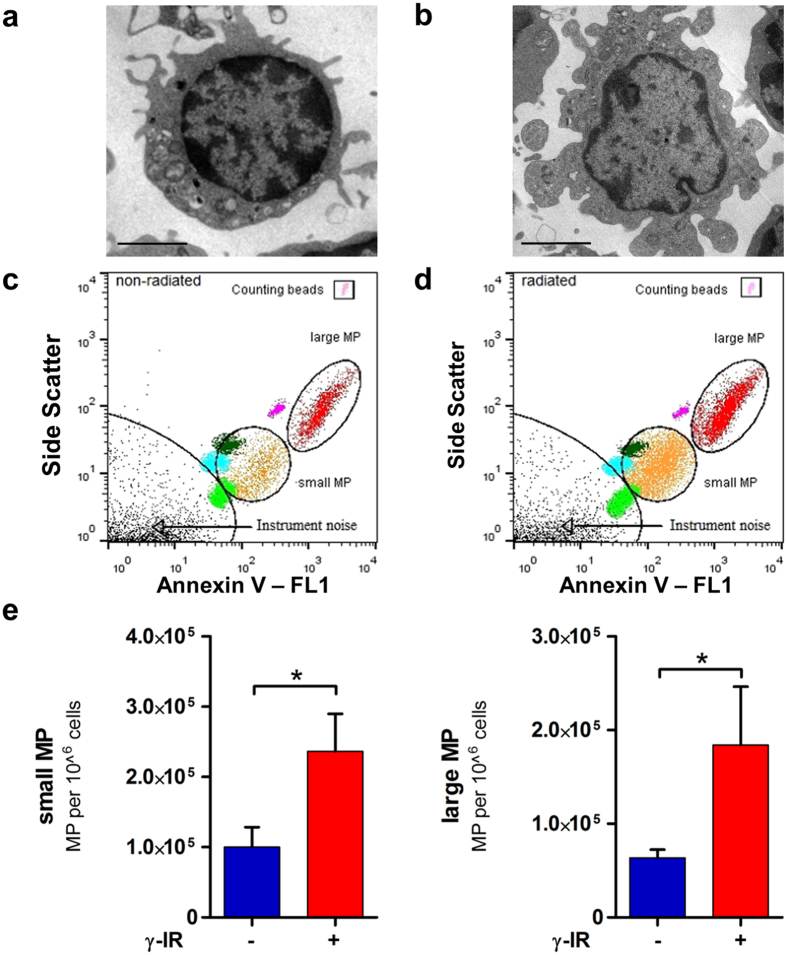
Ionizing radiation induces the release of microparticles. (*Left*) Non-irradiated and (*right*) irradiated PBMCs were cultured for 20 h and then subjected to electron microscopy and FACS analysis. (**a**) Image of non-irradiated PBMC shows a largely intact plasma membrane and cell nucleus with minor plasma membrane shedding. Scale bar = 2.5 μm (**b**) Image of irradiated PBMC shows cellular shrinking, plasma membrane fragmentation, and chromatin condensation. (**c**,**d**) FACS quantification of the absolute number of microparticles released from PBMCs. Microparticles were purified from 1 mL CM containing 25 * 10^6^ PBMCs with a serial centrifugation protocol, and the absolute number of vesicles was calculated with control counting beads. Representative FACS analysis results are shown for (**c**) non-irradiated and (**d**) irradiated PBMCs; the counting beads are indicated on the upper right sides. The scaling beads are highlighted in light green, light blue, dark green, and pink; diameters range from 0.16 μm to 0.5 μm. Two distinct sizes of microparticles are highlighted in orange (small microparticles) and red (large microparticles). (**e**,**f**) Quantitative analyses of non-irradiated (−) and irradiated (+) samples show that irradiation induced release of both (**e**) small and (**f**) large microparticles. n = 3. *p < 0.05

To extend our analysis, we investigated the content of extracellular vesicles present in the CM of non-irradiated and irradiated PBMCs. We first performed TEM imaging of non-irradiated and irradiated PBMCs after 20 h of culture. Non-irradiated PBMCs showed large nuclei with scant cytoplasm (Fig. 4a). Irradiated PMBCs showed dissolution of the cell membrane and more debris between cells (Fig. 4b); these findings suggested that irradiation caused cell fragmentation.

Apoptosis is known to induce shedding of plasma membrane microparticles^36^,^37^. Therefore, we investigated whether irradiation stimulated microparticle release. We isolated microparticles from CM at 20 h after irradiation with differentiated centrifugation procedures. Microparticles were stained with annexin V and detected with FACS analysis, based on SSC/FL1 characteristics. The vesicle diameters ranged from 0.2 to 1 μm, which was typical of microparticles. Filtration of CM through 0.2-μm pore filters removed >99.9% of microparticles, but exosomes were not removed (data not shown). We used counting beads to quantify absolute numbers of microparticles. Representative FACS images of microparticles obtained from non-irradiated and irradiated PBMCs are shown in Fig. 4c,d, respectively. Irradiation induced the release of both small and large microparticles (non-irradiated: 100106 ± 56934 small microparticles/10^6^ cells vs. irradiated 236583 ± 106347 small microparticles/10^6^ cells; p = 0.039; Fig. 4e; non-irradiated 63610 ± 17769 large microparticles/10^6^ cells vs. irradiated 184091 ± 62184 large microparticles/10^6^ cells; p = 0.045; Fig. 4f). Particle diameters were between 0.2 and 0.5 μm.

### Ionizing radiation induces release of exosomes

**Figure 5:**
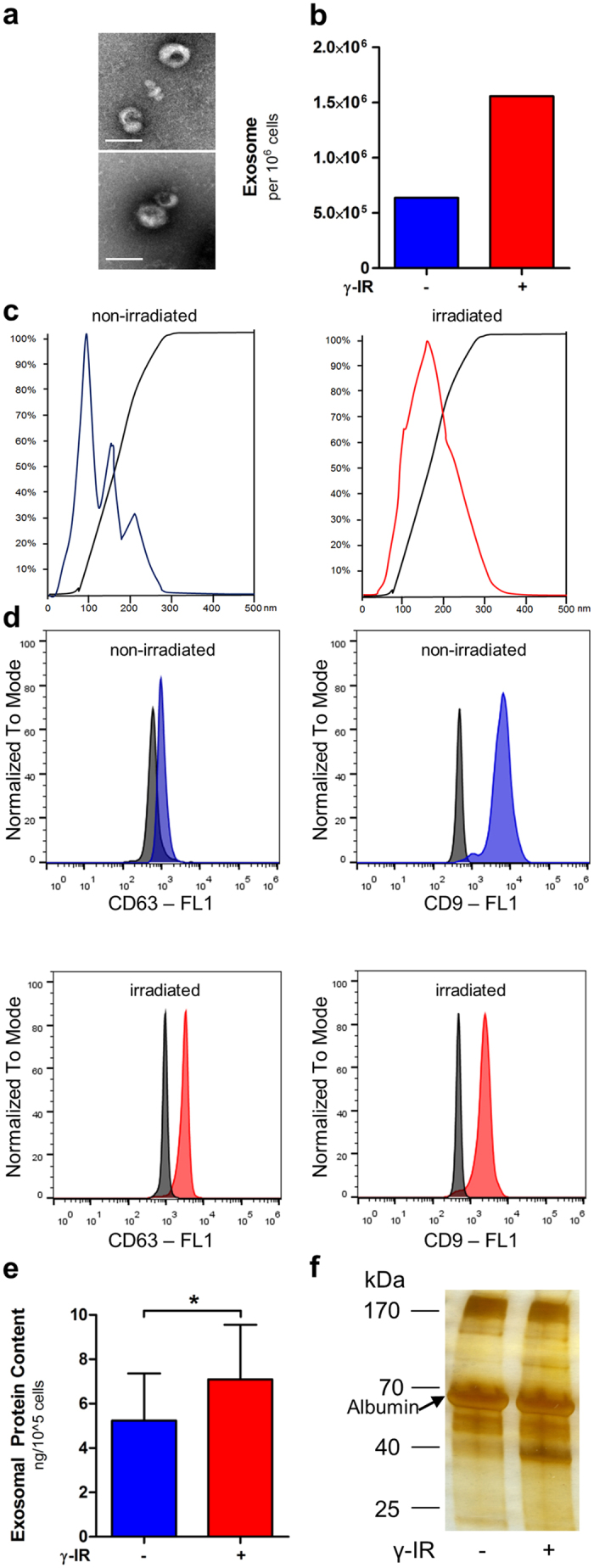
Ionizing radiation induces exosome release and modulates protein content. Exosomes isolated from CM of 25 × 10^6^ non-irradiated (−) or irradiated (+) PBMCs were qualitatively analyzed with transmission electron microscopy (TEM). (**a**) TEM images confirm typical size and shape of exosomes derived from non-irradiated (*top*) and irradiated (*bottom*) PBMCs. Scale bar = 100 nm (**b**) NanoSight analysis shows absolute number of exosomes released per cell at 20 h after cultivation was higher in CM of irradiated compared to the CM from non-irradiated PBMCs. (**c**) Size distribution of exosomes: The mean size ± SD of exosomes was 143 nm ± 56 nm or 177 nm ± 63 nm from non-irradiated and irradiated cells, respectively. The three peaks in the graph indicate that exosomes clumped together during analysis. (**d**) FACS analysis of CD63 and CD9 exosome markers. Exosomes were coupled to CD63 marker beads and labeled with either CD63 or CD9 antibodies. Isotope antibodies served as a negative control. Exosomes from non-irradiated (*top*) and irradiated (*bottom*) PBMCs were positive for both CD63 (*left*) and CD9 (*right*); thus, the purification procedure was sufficient to isolate exosomes. (**e**) Exosomes were lysed in SDS buffer and total protein content was quantified with the Bradford assay. The total protein content was significantly higher in exosomes from irradiated (+) PBMCs than in exosomes from non-irradiated (−) PBMCs. Data are given as mean ± SD of exosome protein concentration in ng per 10^5^ cells. Four individual experiments were performed; *p < 0.05. (**f**) Proteins were separated on SDS gels and stained with silver stain. The large band at 66 kDa depicts the albumin fraction which represents the most abounded protein. Other proteins were differentially expressed in exosomes from irradiated and non-irradiated PBMCs (e.g., band at 37 kDa). One representative experiment of three is shown.

The presence of exosomes in the CM was verified by TEM, FACS, and NanoSight technology. For TEM visualization, exosomes were isolated from CM of irradiated and non-irradiated PBMCs at 20 h of culture with an ultracentrifugation protocol. In negative-stained TEM images, purified vesicles had an approximate diameter of 100 nm, and they were cup-shaped, characteristic of exosomes (Fig. 5a). NanoSight was used to quantify the absolute number of vesicles in the CM of 25 * 10^6^ cells. CM samples derived from irradiated and non-irradiated PBMCs of four donors were pooled to improve statistical analysis. The number of exosomes was 3-fold higher in the CM from irradiated PBMCs (15 * 10^5^ exosomes/10^5^cell) than in the CM from non-irradiated PBMCs (6 * 10^5^ exosomes/10^5^cell) (Fig. 5b). The number of released exosomes correlated with the number of apoptotic cells as shown in supplementary Figure S1. The mean size ± SD of exosomes was 177 nm ± 63 nm from irradiated cells and 143 nm ± 56 nm from non-irradiated cells (Fig. 5c).

For FACS analysis, exosomes were further purified with an anti-CD63 dynabead isolation procedure. Vesicles were separated based on positive detection of the exosome markers, CD63 and CD9 (Fig. 5d). In addition, the protein content in the exosome fraction was significantly higher in irradiated cells than in non-irradiated cells (Fig. 5e).

To determine whether irradiation induced changes in exosome protein content, we analyzed anti-CD63-isolated exosomes, pooled from four independent PBMC preparations, on SDS-PAGE gels. The separated proteins were visualized with silver stain. Figure 5f shows that several bands were differentially expressed in exosomes isolated from irradiated and non-irradiated PBMCs. In addition, we used 2D-difference gel electrophoresis to analyze the differentially expressed proteins in more detail. Supplementary Fig. S5 shows that several protein spots (circled in white) were only detected in the lysates of exosomes purified from irradiated PBMCs; this result suggested that irradiation induced changes in exosome protein content.

### Proteins and exosomes in conditioned media are biologically active

**Figure 6:**
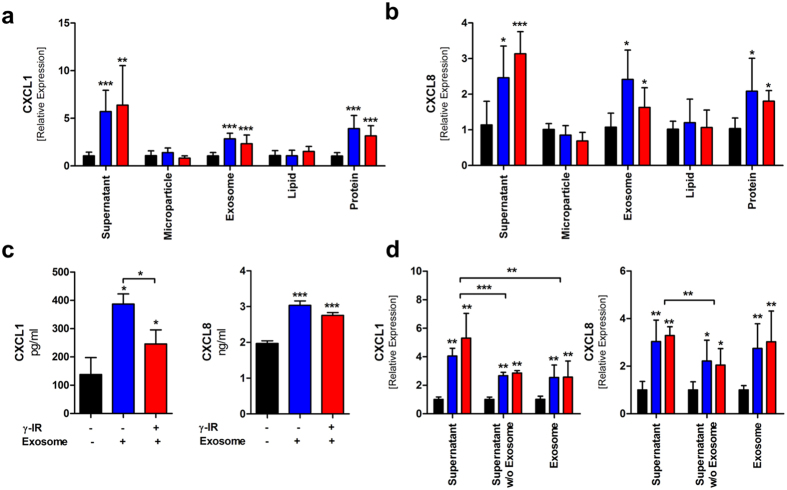
Exosomes and proteins stimulate CXCL1 and CXCL8 expression. Fibroblast gene expression of (**a**) CXCL1 and (**b**) CXCL8 was measured relative to B2M expression (control). Fibroblasts were stimulated with control cell culture media (black bars) or stimulated with total CM (supernatant) or the indicated CM fractions. CM was collected from irradiated (red bars) or non-irradiated (blue bars) PBMCs. RNA was isolated 6 h after cell stimulation. (**c**) ELISA results show CXCL1 and CXLC8 protein contents in the supernatant of fibroblasts after stimulation with exosomes purified from 2,5 * 10^6^ irradiated or non-irradiated PBMCs. Fibroblast cell culture media was harvested 6 h after stimulation and ELISA were performed. (**d**) Exosomes were removed from CM via ultracentrifugation (Supernatant w/o Exosome). Fibroblasts were stimulated with CM, exosome free CM and purified exosomes. Removal of exosomes from CM attenuated it´s capacity to induce CXCL1 and CXCL8 expression. Exosomes and exosome free CM induced CXCL1 and CXCL8 expression in a comparable manner. There were no significant differences between CM and exosomes from non-irradiated and irradiated PBMCs. Bars represent the means ± SD of two (**a–c**) or three experiments (**d**), each performed in triplicate; *p < 0.05; **p < 0.01; ***p < 0.001.

Previous studies have shown that the CM of irradiated and non-irradiated PBMCs promoted angiogenesis and wound healing *in vivo* and stimulated the migration and activation of FBs and KCs^10^. Here, we performed *in vitro* assays to investigate the biological effects of distinct components of the CM. First, primary human FBs were stimulated either with total CM (supernatant) or with different CM fractions, derived from 2.5 * 10^6^ irradiated and non-irradiated PBMCs resuspended in 1 mL basal media without growth factors. As expected, CXCL1 and CXCL8 expression were induced in response to stimulation with total CM (Fig. 6a,b). In addition, the expression of both cytokines increased when stimulated with exosomes or the protein fraction of CM derived from both irradiated and non-irradiated PBMCs. No significant differences were observed between non-irradiated PBMCs and irradiated PBMCs. Next, we isolated CXCL1 and CXCL8 proteins from the CM of FBs and measured them with ELISA. We found that secretion of these cytokines in FB was significantly induced by PBMC exosomes (Fig. 6c). Exosomes from non-irradiated PBMCs induced CXLC1 release in a significant higher amount as compared to exosomes of irradiated PBMCs. Exosome free CM showed an attenuated induction of CXCL1 and CXCL8 expression in FB as compared to complete CM (Fig. 6d).

When the same experiments were performed with primary human KCs, we again found that the total CM, the exosomes, and the protein fraction could stimulate CXCL1 and CXLC8 expression (supplementary Fig. S6A,B). The CM and protein fraction of irradiated PBMCs led to a significant stronger induction of gene expression than those of non-irradiated samples.

**Figure 7:**
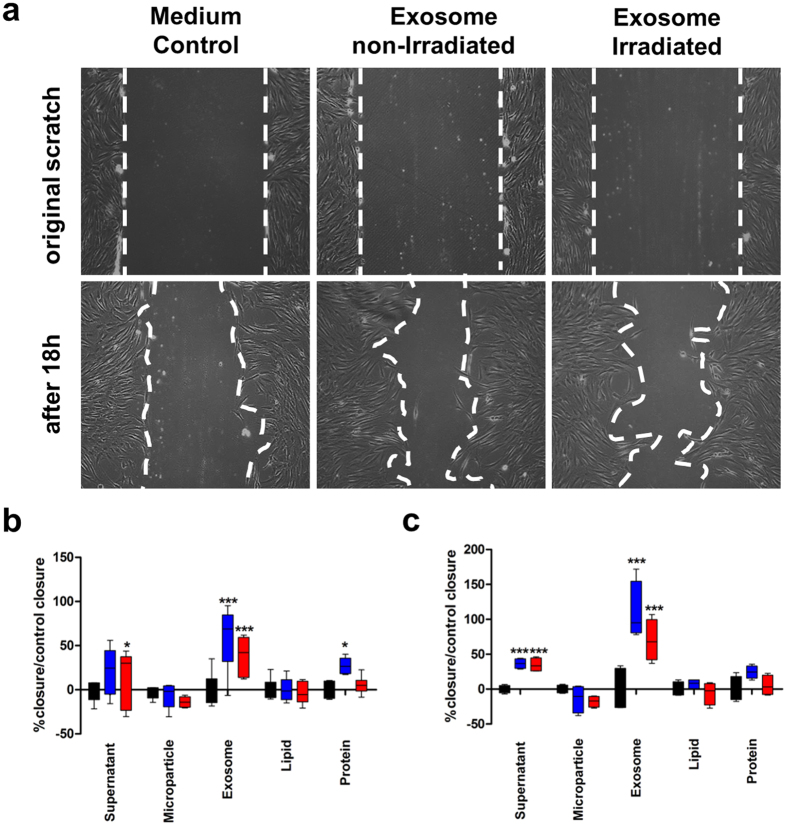
Exosomes and CM proteins enhance cell migration. (**a**), *top*) Fibroblast monolayers were scratched (cleared areas outlined in white dashed lines) to simulate a wound. Cultures were untreated or treated with exosome preparations from non-irradiated or irradiated PBMCs. (*Bottom*) After 18 h, fibroblast proliferation began to close the wound in (*left*) untreated and (*middle*, *right*) treated cultures. Treatment with PBMC-derived exosomes accelerated wound closure. 10 fold magnification (**b**,**c**) Wound areas were measured in 8 scratches after (**b**) 24 h and (**c**) 48 h. The percentage of closure compared to closure in control (untreated) was calculated. There were no significant differences between non-irradiated and irradiated PBMCs detectable. Data are expressed as the mean ± SD of two independent experiments. *p < 0.05; **p < 0.01, ***p < 0.001.

We performed scratch assays in FB cultures to examine simulated wound healing. We found that closure of the scratch wounds was significantly enhanced after 18 (Fig. 7a,b), and 48 h (Fig. 7c), when cells were cultured in the presence of total CM, exosomes, or the protein fraction of CM isolated from 2.5 * 10^6^ PBMCs resuspended in 1 mL basal media without growth factors. Scratch assays were also performed with KCs with similar results (supplementary Fig. S7A–C). These results supported the notion that exosomes and proteins were the main biologically active components of CM. In these experiments, the lipid fraction was the water-soluble fraction derived from extracellular vesicles; it should be noted that exosomes contain lipids that are also biologically active.

### Stability of CM components and a large animal model of myocardial infarction

Strict legal requirements must be met for therapeutic use of biological materials in humans. The experimental settings used in basic science and pre-clinical studies for testing *in vitro* and *in vivo* effects of substances typically must be altered for clinical studies, due to legal requirements. With these restrictions in mind, we produced and handled the CM according to good manufacturing practice (GMP) guidelines. Therefore, GMP-compliant CM could be used for cell-free therapy in humans. We compared the biological efficiency of these GMP-compliant and the experimentally-prepared supernatants. First, the quantity and quality of biological components derived from GMP-compliant CM samples were comparable to those of experimental CM samples (supplementary Fig. S8A–E). The lipid analysis revealed that the GMP-compliant CM was enriched in oxidized phospholipids, similar to those detected in the experimental CM. However, the GMP-compliant CM did not contain microparticles, because it had to be filtered through 0.2-μm filters to eliminate all particles with diameters greater than 0.2 μm. However, the quantity and quality of CM exosomes were comparable between the experimental and GMP-compliant samples; this similarity suggested that the exosomes were relatively resistant to GMP procedures. Selected proteins were analyzed with ELISA, and we found comparable protein concentrations.

Furthermore, we compared the experimental CM and GMP-compliant CM for their *in vivo* effects in an experimental model of AMI in domestic pigs. In previous studies, we showed that, after coronary artery ligation, myocardial damage was attenuated when the CM of irradiated PBMCs was injected 45 min after the onset of ischemia^6^. As shown in Table 1, the experimental CM and GMP-compliant CM samples were comparable in their capacity to attenuate ischemic damage following coronary artery occlusion. Both treatment groups showed improved cardiac output and reduced infarct areas compared to controls at 30 days after infarction.

## Discussion

In the present study, we investigated the paracrine factors released from non-irradiated and irradiated human PBMCs and explored the biologically active components. We demonstrated that irradiation quantitatively and qualitatively changed the release of proteins, lipids, and extracellular vesicles in human PBMCs. Subsequently, we showed that two biological components in CM, the protein and exosome fractions, exerted the majority of proliferative and stimulatory effects observed in selected *in vitro* experiments. Moreover, our analysis revealed that the biological activity of CM in experimental AMI was not influenced by GMP procedures.

Cell-based therapies have shown promise for treating multiple diseases related to hypoxia-induced inflammation (e.g., AMI, stroke)^38^. In the last decade, results from randomized controlled trials have shown that the infusion of cells derived from different sources improved clinical endpoints^39^. In the initial stages of stem cell therapy research, it was thought that the observed effects were due to the direct interactions between donor and host cells. However, a growing body of evidence has given rise to the current notion that the beneficial effects are mediated by paracrine signaling, rather than direct interactions^4^,^40^.

A growing number of studies have shown that paracrine factors can modulate the host immune system, improve survival after myocardial infarction and ischemic stroke, and attenuate neurological disorders^14^,^17^,^41^,^42^. Most studies have focused on evaluating proteins as mediators of this paracrine capacity. However, conditioned medium (CM) contains proteins, lipids, and extracellular vesicles^15^. To our knowledge, no previous study has investigated these biological components in the field of regenerative medicine.

In this study, we quantitatively compared proteins, lipids, and extracellular vesicles present in the CM of non-irradiated and irradiated PBMCs. We used stressed PBMCs, because accumulating evidence has indicated that paracrine activity could be stimulated by hypoxia^22^,^23^, cell starvation^43^, and apoptosis^5^,^6^,^7^,^8^,^9^,^12^,^44^.

First, we implemented a bioinformatics-based analysis of the secretome designed to identify secreted proteins^45^,^46^. We identified 213 genes that encoded secreted proteins, which were upregulated in response to irradiation. In contrast, non-irradiated PBMCs showed upregulation of only 176 transcripts; this difference suggested that irradiation triggered additional secretion. The 213 genes upregulated in irradiated PBMCs showed an enrichment of proteins involved in biological processes related to angiogenesis, cell proliferation, and cytokine signaling. Interestingly, a previous study that analyzed proteins in a stem cell secretome revealed a similar enrichment in proteins involved in biological processes and pathways^47^. In another study, a proteomic analysis of a mesenchymal stem cell secretome showed enrichment in genes annotated with biological processes involved in angiogenesis, blood vessel morphogenesis, chemotaxis, wound response, and stress response, among others^48^. Those results were partly comparable to the results of our study. In addition to the 213 proteins that we identified with stringent bioinformatics analysis, other proteins that were undetected with our methods might have been present in the CM, due to passive release from dying cells. Moreover, our method did not identify VEGF or PAI-1, two proteins involved in the regulation of angiogenesis, which are known to be present at high concentrations in the CM of irradiated PBMCs^6^. However, we identified several other factors in the CM of stressed PBMCs that have known effects in the field of regenerative medicine^47^. For example, adrenomedullin was released in response to irradiation. According to Okumara *et al*., adrenomedullin has pro-angiogenic properties, inhibits cardiac fibrosis, and exerts cardio-protective effects^49^. Also, administration of growth differentiation factor 15 was shown to attenuate ischemia reperfusion damage^50^. Insulin like growth factor exerted anti-apoptotic, pro-angiogenic, and cell proliferative activities^51^. In addition to these factors with known reparative potential, we identified several other factors in this study. For example, we showed that stressed PBMCs expressed MMP9, VEGFA, TIMP-1, TSP-1, PDGF, FGF, and several other cyto- and chemokines. In accordance with our bioinformatics data, we found that purified CM proteins could induce cell migration and CXCL1 and CXCL8 expression in FBs and KCs, which are both involved in wound healing and angiogenesis^52^.

We also studied the presence and activity of other biological components in PBMC-derived CM, including lipids and extracellular vesicles. Extracellular vesicles (comprising exosomes and microparticles) are currently gaining interest, because they have emerged as a new mechanism for intercellular communication. Extracellular vesicles contain mRNAs, miRNAs, and proteins^20^,^21^. By direct horizontal transfer of mRNA, miRNAs, and proteins, extracellular vesicles have been shown to stimulate the regenerative capacity of injured tissues^53^. Although the exact mechanism by which extracellular vesicles exert their regenerative capacity is currently debated, accumulating evidence has indicated that extracellular vesicles increase endothelial cell proliferation, induce angiogenesis, modulate extracellular matrix interactions, and modulate immune activities (reviewed in^17^).

In the present study, exosomes derived from irradiated as well as from non-irradiated PBMCs induced CXCL1 and CXCL8 expression and enhanced cell migration in primary human skin cells, but microparticles did not show any activities *in vitro*. We were able to show that exosomes and proteins are the two main biological components that stimulate CXCL1 and CXCL8 gene expression. The strongest effects were seen for unprocessed CM containing exosomes and proteins. The effect of the individual components alone was less marked as compared to unprocessed CM, indicating that there are additive or synergistic effects, respectively. Removing exosomes from the CM attenuated the biological activity of CM comparable to that observed with the protein fraction. These data support our hypothesis that exosomes and proteins are the two main biological components that induce pro-angiogenic gene expression in human FB and KCs.

We did not observe any significant differences in gene expression between exosomes and proteins derived from non-irradiated or irradiated PBMCs when we stimulated FB. However, KCs were more reactive to components of irradiated PBMCs. Our data indicate that both PBMCs conditions have paracrine effects showing slightly different capacity depending of the cell type and experimental setup.

Furthermore, we showed that irradiated cells released more extracellular vesicles than non-irradiated cells. This data are in line with others showing that irradiation induced exosome release in astrocytes and glioblastoma cell in a does dependent manner^54^. Although electron microscopy evidenced the typical size and cup-shape of exosomes (~100 nm), NanoSight analysis revealed a mean diameter of ~150 nm, which was most likely a result of exosome-conglomeration. Our difference-gel electrophoresis analysis of lysed exosomes derived from irradiated and non-irradiated PBMCs showed that several proteins were differentially expressed. These findings indicated that irradiation induced exosome secretion and altered exosome protein content. However, a more focused investigation of the function of these proteins will require more sophisticated experiments; therefore, it was beyond the scope of the present study. We speculate that irradiation may increase CM exosome secretion through p53 signaling, based on the facts that irradiation activates the p53 pathway^31^, and this pathway was shown to be involved in regulating exosome release^55^,^56^. However, it remains to be determined how irradiation regulates the changes in exosome protein content. To further analyze whether irradiation induced qualitative changes of exosomal proteins we performed 1D and 2D gel electrophoresis. We identified several proteins which were differentially present in exosomes from non-irradiated and irradiated PBMCs. Of note although we got three times more exosomes from irradiated cell, the total protein content differed only marginal between exosomes from non-irradiated and irradiated cells. This could be explained by the fact that during exosome preparation high amounts of albumin get separated^57^ which interferes with the measurement of exosomal proteins (Fig. 5f). Sirois and colleagues showed that exosomes released from apoptotic endothelial cells exerted anti-apoptotic activity in vascular smooth muscle cells by activating ERK 1/2 receptors^43^. In a previous study, Lichtenauer *et al*. showed that the secretome of apoptotic PBMCs displayed similar biochemical effects on human cardiomyocytes; they promoted resistance to apoptosis and activated ERK1/2 receptors^6^. It is tempting to speculate that these effects might be mediated by exosomes. Further studies are planned to investigate these interesting questions in a large animal model of AMI.

Our analyses of GMP-compliant CM prepared from irradiated PBMCs showed convincing evidence that the CM retained potency under restricted conditions. This was the first proof-of-principle to test whether the pathogen reduction process would abolish the biological efficiency of the CM in a standardized experimental setup. First, we tested the total CM (not fractionated) from irradiated PBMCs. We compared CM prepared with the experimental methods to that prepared with GMP-compliant methods. Both of these CM preparations had comparable biological effects in a model of AMI, compared to CM from non-irradiated PBMCs. These effects were most likely provoked by an interplay among several factors (e.g., proteins and exosomes), rather than by a single component, and these factors were retained in the GMP methods.

This was the first study to evaluate the effect of irradiation on the oxidized lipid content of the secretome, which includes microparticles, exosomes, and soluble lipids. We investigated irradiation-induced changes by performing lipidomics on CM samples from irradiated and non-irradiated PBMCs. We focused on oxPCs. High pressure lipid chromatography-tandem MS analysis of PCs showed that irradiation promoted the formation of oxidized lipid species with pro-angiogenic and immunomodulatory properties (reviewed in^16^). Our lipidomics protocol enabled detection of a large number of oxidation products derived from the most abundant cell membrane phospholipids^30^. We found that CM obtained from irradiated PBMCs contained significantly higher concentrations of specific oxPCs than CM from non-irradiated PBMCs. Microparticles represent the largest class of extracellular vesicles, and they are abundantly present in the CM from irradiated PBMCs. PCs are the most abundant lipid class in microparticles^58^; in contrast, exosomes have less phospholipid and more ceramide contents than microparticles. Therefore, we speculated that irradiation-induced changes in oxidation products might be predominantly mediated by the oxidation of PCs incorporated in microparticles. However, although oxPCs were previously shown to exert biological activity^30^,^32^,^59^, induce expression of CXCL8, and modulate angiogenesis^60^, we could not detect any *in vitro* effects of soluble CM lipids in our selected experiments. It is tempting to speculate that probably other cell types than those used in this study might be sensitive to oxPC treatments. These findings should stimulate further research with different functional assays to clarify the role of PBMC-derived lipids as paracrine mediators.

In summary, we demonstrated that irradiation induced quantitative and qualitative changes in the secretome of human PBMCs. Irradiated cells expressed higher amounts of pro-angiogenic proteins, extracellular vesicles, and oxidized phospholipids than non-irradiated cells. In selected *in vitro* assays with primary human FBs and KCs, we showed that the two main biologically active components of CM were in the fractions that contained either proteins or exosomes. Validated, viral-cleared GMP-compliant, PBMC secretomes displayed cardioprotective effects comparable to those displayed with experimental-grade CM in an *in vivo* model of AMI. This study provided a basis for the development of cell-free therapies in the field of regenerative medicine.

## Additional Information

**How to cite this article**: Beer, L. *et al*. Analysis of the Secretome of Apoptotic Peripheral Blood Mononuclear Cells: Impact of Released Proteins and Exosomes for Tissue Regeneration. *Sci. Rep*. **5**, 16662; doi: 10.1038/srep16662 (2015).

## Supplementary Material

Supplementary Information

## Figures and Tables

**Table 1:** Cardic MRI evaluation 3 and 30 days after AMI.

	Parameters	Medium Control (n = 7)	CM (1,5 × 10^9^; n = 4)	CM + PR (1,5 × 10^9^; n = 6)	
After 3 days	Weight (kg)	31.9 ± 0.9	32.0 ± 1.2	34.2 ± 0.5	n.s.
	Age (days)	90 ± 0	90 ± 0	90 ± 0	n.s.
	LVEDV (ml)	67.6 ± 2.8	75.6 ± 2.2	83.0 ± 4.0*	*
	LVESV(ml)	38.4 ± 2.5	47.4 ± 1.7*	48.7 ± 4.3	n.s.
	LVSV (ml)	29.2 ± 1.3	28.3 ± 2.2	34.3 ± 2.0*	n.s.
	LVEF (%)	43.4 ± 1.9	37.3 ± 2.2	41.7 ± 2.9	n.s.
	HR/min	111 ± 6	87 ± 9*	77 ± 3**	**
	CO (l/min)	3.2 ± 0.1	2.4 ± 0.1*	2.6 ± 0.2*	**
	CI (l/min/m2)	3.6 ± 0.1	3.1 ± 0.3	3.3 ± 0.2	n.s.
	Infarct %	18.2 ± 1.7	13.1 ± 2.8	12.3 ± 1.9*	n.s.
After 30 days	Weight (kg)	39.4 ± 0.5	50.0 ± 1.8***	55.7 ± 0.7***	***
	Age (days)	120 ± 0	120 ± 0	120 ± 0	n.s.
	LVEDV (ml)	54.8 ± 4.1	107.5 ± 6.7***	102.5 ± 6.0***	***
	LVESV(ml)	32.9 ± 4.0	65.5 ± 3.4***	53.9 ± 4.3**	***
	LVSV (ml)	21.8 ± 1.8	42.0 ± 4.4**	48.6 ± 2.9***	***
	LVEF (%)	40.5 ± 3.6	38.9 ± 2.3	47.6 ± 2.1	n.s.
	HR/min	114 ± 7	123 ± 5	109 ± 3	n.s.
	CO (l/min)	2.4 ± 0.1	5.1 ± 0.4***	5.3 ± 0.3 ***	***
	CI (l/min/m2)	2.5 ± 0.1	5.0 ± 0.3***	4.7 ± 0.3***	***
	Infarct %	12.6 ± 1.4	9.8 ± 0.6	8.2 ± 1.7	n.s.

Three and 30 days after ischaemia/reperfusion injury, MRI was conducted and parameters of cardiac function were obtained from pigs treated with unprocessed CM (CM) and pathogen reduced CM (PR+CM) and from control animals.

LVEDD left ventricular end-diastolic diameter, LVESD left ventricular end-systolic diameter, LVSV left ventricular stroke volume, LVEF left ventricular ejection fraction, HR heart rate, CI cardiac index, CO cardiac output, ns no significance versus control.

*p < 0.05 vs control.

**p < 0.01 vs control.

***p < 0.001 vs control.

## References

[b1] Ptaszek L. M., Mansour M., Ruskin J. N. & Chien K. R. Towards regenerative therapy for cardiac disease. Lancet 379, 933–942 (2012). 22405796 10.1016/S0140-6736(12)60075-0

[b2] Gurtner G. C., Werner S., Barrandon Y. & Longaker M. T. Wound repair and regeneration. Nature 453, 314–321 (2008). 18480812 10.1038/nature07039

[b3] Sahoo S. & Losordo D. W. Exosomes and cardiac repair after myocardial infarction. Circ Res 114, 333–344 (2014). 24436429 10.1161/CIRCRESAHA.114.300639

[b4] Gnecchi M. . Paracrine action accounts for marked protection of ischemic heart by Akt-modified mesenchymal stem cells. Nat Med 11, 367–368 (2005). 15812508 10.1038/nm0405-367

[b5] Lichtenauer M. . Intravenous and intramyocardial injection of apoptotic white blood cell suspensions prevents ventricular remodelling by increasing elastin expression in cardiac scar tissue after myocardial infarction. Basic Res Cardiol 106, 645–655 (2011). 21416207 10.1007/s00395-011-0173-0 PMC3105227

[b6] Lichtenauer M. . Secretome of apoptotic peripheral blood cells (APOSEC) confers cytoprotection to cardiomyocytes and inhibits tissue remodelling after acute myocardial infarction: a preclinical study. Basic Res Cardiol 106, 1283–1297 (2011). 21952733 10.1007/s00395-011-0224-6 PMC3228946

[b7] Hoetzenecker K. . Secretome of apoptotic peripheral blood cells (APOSEC) attenuates microvascular obstruction in a porcine closed chest reperfused acute myocardial infarction model: role of platelet aggregation and vasodilation. Basic Res Cardiol 107, 292 (2012). 22899170 10.1007/s00395-012-0292-2 PMC3442164

[b8] Hoetzenecker K. . Mononuclear cell secretome protects from experimental autoimmune myocarditis. Eur Heart J 36, 676–685 (2015). 23321350 10.1093/eurheartj/ehs459 PMC4359357

[b9] Pavo N. . Long-acting beneficial effect of percutaneously intramyocardially delivered secretome of apoptotic peripheral blood cells on porcine chronic ischemic left ventricular dysfunction. Biomaterials 35, 3541–3550 (2014). 24439416 10.1016/j.biomaterials.2013.12.071

[b10] Mildner M. . Secretome of peripheral blood mononuclear cells enhances wound healing. PLoS One 8, e60103 (2013). 23533667 10.1371/journal.pone.0060103 PMC3606336

[b11] Cervio M. . gamma-Irradiated cord blood MNCs: Different paracrine effects on mature and progenitor endothelial cells. Microvasc Res 94, 9–16 (2014). 24788073 10.1016/j.mvr.2014.04.009

[b12] Altmann P. . Secretomes of apoptotic mononuclear cells ameliorate neurological damage in rats with focal ischemia. F1000Res 3, 131 (2014). 25383184 10.12688/f1000research.4219.1 PMC4215751

[b13] Korf-Klingebiel M. . Bone marrow cells are a rich source of growth factors and cytokines: implications for cell therapy trials after myocardial infarction. Eur Heart J 29, 2851–2858 (2008). 18953051 10.1093/eurheartj/ehn456

[b14] Korf-Klingebiel M. . Myeloid-derived growth factor (C19orf10) mediates cardiac repair following myocardial infarction. Nat Med 21, 140–149 (2015). 25581518 10.1038/nm.3778

[b15] Lauber K. . Apoptotic cells induce migration of phagocytes via caspase-3-mediated release of a lipid attraction signal. Cell 113, 717–730 (2003). 12809603 10.1016/s0092-8674(03)00422-7

[b16] Bochkov V. N. . Generation and biological activities of oxidized phospholipids. Antioxid Redox Signal 12, 1009–1059 (2010). 19686040 10.1089/ars.2009.2597 PMC3121779

[b17] De Jong O. G., Van Balkom B. W., Schiffelers R. M., Bouten C. V. & Verhaar M. C. Extracellular vesicles: potential roles in regenerative medicine. Front Immunol 5, 608 (2014). 25520717 10.3389/fimmu.2014.00608 PMC4253973

[b18] Xin H. . Systemic administration of exosomes released from mesenchymal stromal cells promote functional recovery and neurovascular plasticity after stroke in rats. J Cereb Blood Flow Metab 33, 1711–1715 (2013). 23963371 10.1038/jcbfm.2013.152 PMC3824189

[b19] Zhang B. . HucMSC-exosome mediated -Wnt4 signaling is required for cutaneous wound healing. Stem Cells 33, 2158–2168 (2014). 24964196 10.1002/stem.1771

[b20] Valadi H. . Exosome-mediated transfer of mRNAs and microRNAs is a novel mechanism of genetic exchange between cells. Nat Cell Biol 9, 654–659 (2007). 17486113 10.1038/ncb1596

[b21] Vlassov A. V., Magdaleno S., Setterquist R. & Conrad R. Exosomes: current knowledge of their composition, biological functions, and diagnostic and therapeutic potentials. Biochim Biophys Acta 1820, 940–948 (2012). 22503788 10.1016/j.bbagen.2012.03.017

[b22] Salomon C. . Exosomal signaling during hypoxia mediates microvascular endothelial cell migration and vasculogenesis. PLoS One 8, e68451 (2013). 23861904 10.1371/journal.pone.0068451 PMC3704530

[b23] Zhang H. C. . Microvesicles derived from human umbilical cord mesenchymal stem cells stimulated by hypoxia promote angiogenesis both *in vitro* and *in vivo*. Stem cells and development 21, 3289–3297 (2012). 22839741 10.1089/scd.2012.0095 PMC3516422

[b24] Brazma A. . Minimum information about a microarray experiment (MIAME)-toward standards for microarray data. Nat Genet 29, 365–371 (2001). 11726920 10.1038/ng1201-365

[b25] Ramskold D., Wang E. T., Burge C. B. & Sandberg R. An abundance of ubiquitously expressed genes revealed by tissue transcriptome sequence data. PLoS Comput Biol 5, e1000598 (2009). 20011106 10.1371/journal.pcbi.1000598 PMC2781110

[b26] Petersen T. N., Brunak S., von Heijne G. & Nielsen H. SignalP 4.0: discriminating signal peptides from transmembrane regions. Nat Methods 8, 785–786 (2011). 21959131 10.1038/nmeth.1701

[b27] Choo K. H., Tan T. W. & Ranganathan S. A comprehensive assessment of N-terminal signal peptides prediction methods. BMC Bioinformatics 10 Suppl 15, S2 (2009). 19958512 10.1186/1471-2105-10-S15-S2 PMC2788353

[b28] Krogh A., Larsson B., von Heijne G. & Sonnhammer E. L. Predicting transmembrane protein topology with a hidden Markov model: application to complete genomes. Journal of molecular biology 305, 567–580 (2001). 11152613 10.1006/jmbi.2000.4315

[b29] Wang J., Duncan D., Shi Z. & Zhang B. WEB-based GEne SeT AnaLysis Toolkit (WebGestalt): update 2013. Nucleic Acids Res 41, W77–83 (2013). 23703215 10.1093/nar/gkt439 PMC3692109

[b30] Gruber F., Bicker W., Oskolkova O. V., Tschachler E. & Bochkov V. N. A simplified procedure for semi-targeted lipidomic analysis of oxidized phosphatidylcholines induced by UVA irradiation. J Lipid Res 53, 1232–1242 (2012). 22414483 10.1194/jlr.D025270 PMC3351830

[b31] Beer L. . High dose ionizing radiation regulates micro RNA and gene expression changes in human peripheral blood mononuclear cells. BMC genomics 15, 814 (2014). 25257395 10.1186/1471-2164-15-814 PMC4182888

[b32] Gruber F. . NF-E2-related factor 2 regulates the stress response to UVA-1-oxidized phospholipids in skin cells. FASEB J 24, 39–48 (2010). 19720622 10.1096/fj.09-133520 PMC2797031

[b33] Beer L. . Bioinformatics approach for choosing the correct reference genes when studying gene expression in human keratinocytes. Exp Dermatol 24, 742–747 (2015). 25980460 10.1111/exd.12759

[b34] Pappinen S. . Comparison of rat epidermal keratinocyte organotypic culture (ROC) with intact human skin: lipid composition and thermal phase behavior of the stratum corneum. Biochim Biophys Acta 1778, 824–834 (2008). 18211819 10.1016/j.bbamem.2007.12.019

[b35] Gruber F. . Photooxidation generates biologically active phospholipids that induce heme oxygenase-1 in skin cells. J Biol Chem 282, 16934–16941 (2007). 17449870 10.1074/jbc.M702523200

[b36] Vion A. C. . Shear stress regulates endothelial microparticle release. Circ Res 112, 1323–1333 (2013). 23536307 10.1161/CIRCRESAHA.112.300818

[b37] Distler J. H. . The release of microparticles by apoptotic cells and their effects on macrophages. Apoptosis 10, 731–741 (2005). 16133865 10.1007/s10495-005-2941-5

[b38] Passier R., van Laake L. W. & Mummery C. L. Stem-cell-based therapy and lessons from the heart. Nature 453, 322–329 (2008). 18480813 10.1038/nature07040

[b39] Otto W. R. & Wright N. A. Mesenchymal stem cells: from experiment to clinic. Fibrogenesis Tissue Repair 4, 20 (2011). 21902837 10.1186/1755-1536-4-20 PMC3182886

[b40] Hsiao S. T. . Comparative analysis of paracrine factor expression in human adult mesenchymal stem cells derived from bone marrow, adipose, and dermal tissue. Stem cells and development 21, 2189–2203 (2012). 22188562 10.1089/scd.2011.0674 PMC3411362

[b41] Kumar A. H. & Caplice N. M. Clinical potential of adult vascular progenitor cells. Arterioscler Thromb Vasc Biol 30, 1080–1087 (2010). 20453166 10.1161/ATVBAHA.109.198895

[b42] Sahoo S. . Exosomes from human CD34(+) stem cells mediate their proangiogenic paracrine activity. Circ Res 109, 724–728 (2011). 21835908 10.1161/CIRCRESAHA.111.253286 PMC3201702

[b43] Sirois I. . Caspase-3-dependent export of TCTP: a novel pathway for antiapoptotic intercellular communication. Cell Death Differ 18, 549–562 (2011). 20966960 10.1038/cdd.2010.126 PMC3131994

[b44] Ankersmit H. J. . Irradiated cultured apoptotic peripheral blood mononuclear cells regenerate infarcted myocardium. Eur J Clin Invest 39, 445–456 (2009). 19397690 10.1111/j.1365-2362.2009.02111.x

[b45] Mukherjee P. & Mani S. Methodologies to decipher the cell secretome. Biochim Biophys Acta 1834, 2226–2232 (2013). 23376189 10.1016/j.bbapap.2013.01.022 PMC3652893

[b46] Caccia D., Dugo M., Callari M. & Bongarzone I. Bioinformatics tools for secretome analysis. Biochim Biophys Acta 1834, 2442–2453 (2013). 23395702 10.1016/j.bbapap.2013.01.039

[b47] Ranganath S. H., Levy O., Inamdar M. S. & Karp J. M. Harnessing the mesenchymal stem cell secretome for the treatment of cardiovascular disease. Cell stem cell 10, 244–258 (2012). 22385653 10.1016/j.stem.2012.02.005 PMC3294273

[b48] Sze S. K. . Elucidating the secretion proteome of human embryonic stem cell-derived mesenchymal stem cells. Mol Cell Proteomics 6, 1680–1689 (2007). 17565974 10.1074/mcp.M600393-MCP200

[b49] Okumura H. . Adrenomedullin infusion attenuates myocardial ischemia/reperfusion injury through the phosphatidylinositol 3-kinase/Akt-dependent pathway. Circulation 109, 242–248 (2004). 14691041 10.1161/01.CIR.0000109214.30211.7C

[b50] Wollert K. C. Growth-differentiation factor-15 in cardiovascular disease: from bench to bedside, and back. Basic Res Cardiol 102, 412–415 (2007). 17546529 10.1007/s00395-007-0662-3

[b51] Haider H., Jiang S., Idris N. M. & Ashraf M. IGF-1-overexpressing mesenchymal stem cells accelerate bone marrow stem cell mobilization via paracrine activation of SDF-1alpha/CXCR4 signaling to promote myocardial repair. Circ Res 103, 1300–1308 (2008). 18948617 10.1161/CIRCRESAHA.108.186742

[b52] Spiekstra S. W., Breetveld M., Rustemeyer T., Scheper R. J. & Gibbs S. Wound-healing factors secreted by epidermal keratinocytes and dermal fibroblasts in skin substitutes. Wound Repair Regen 15, 708–717 (2007). 17971017 10.1111/j.1524-475X.2007.00280.x

[b53] Yu B. . Exosomes secreted from GATA-4 overexpressing mesenchymal stem cells serve as a reservoir of anti-apoptotic microRNAs for cardioprotection. Int J Cardiol 182C, 349–360 (2014). 25590961 10.1016/j.ijcard.2014.12.043 PMC4382384

[b54] Arscott W. T. . Ionizing radiation and glioblastoma exosomes: implications in tumor biology and cell migration. Transl Oncol 6, 638–648 (2013). 24466366 10.1593/tlo.13640 PMC3890698

[b55] Yu X., Harris S. L. & Levine A. J. The regulation of exosome secretion: a novel function of the p53 protein. Cancer Res 66, 4795–4801 (2006). 16651434 10.1158/0008-5472.CAN-05-4579

[b56] Lespagnol A. . Exosome secretion, including the DNA damage-induced p53-dependent secretory pathway, is severely compromised in TSAP6/Steap3-null mice. Cell Death Differ 15, 1723–1733 (2008). 18617898 10.1038/cdd.2008.104

[b57] Caradec J. . Reproducibility and efficiency of serum-derived exosome extraction methods. Clin Biochem 47, 1286–1292 (2014). 24956264 10.1016/j.clinbiochem.2014.06.011

[b58] Bicalho B., Holovati J. L. & Acker J. P. Phospholipidomics reveals differences in glycerophosphoserine profiles of hypothermically stored red blood cells and microvesicles. Biochim Biophys Acta 1828, 317–326 (2013). 23123566 10.1016/j.bbamem.2012.10.026

[b59] Zhao Y. . Autophagy is induced by UVA and promotes removal of oxidized phospholipids and protein aggregates in epidermal keratinocytes. J Invest Dermatol 133, 1629–1637 (2013). 23340736 10.1038/jid.2013.26

[b60] Bochkov V. N. . Oxidized phospholipids stimulate angiogenesis via autocrine mechanisms, implicating a novel role for lipid oxidation in the evolution of atherosclerotic lesions. Circ Res 99, 900–908 (2006). 16973904 10.1161/01.RES.0000245485.04489.ee

