# Supplementary material for: Analysis of the Secretome of Apoptotic Peripheral Blood Mononuclear Cells: Impact of Released Proteins and Exosomes for Tissue Regeneration
Source: Sci Rep. 2015 Nov 16;5:16662. doi: 10.1038/srep16662 (PMC4645175; doi:10.1038/srep16662)
Supplement: Supplementary Information [file srep16662-s1.pdf]

Supplementary information for:

Analysis of the Secretome of Apoptotic Peripheral Blood Mononuclear Cells: Impact of Released Proteins and Exosomes for Tissue Regeneration

Lucian Beer[1,2], Matthias Zimmermann[1,2], Andreas Mitterbauer[1,2], Adolf Ellinger[3], Florian Gruber[4,5], Marie-Sophie Narzt[4,5], Maria Zellner[6], Mariann Gyöngyösi[7], Sibylle Madlener[8], Elisabeth Simader[1,2]; Christian Gabriel[9], Michael Mildner[4*], Hendrik Jan Ankersmit[1,2*]

[1] Department of Thoracic Surgery, Medical University of Vienna, Austria
[2] Christian Doppler Laboratory for Cardiac and Thoracic Diagnosis and Regeneration, Medical University of Vienna, Austria
[3]Department of Cell Biology and Ultrastructure Research, Center for Anatomy and Cell Biology, Medical University of Vienna, Vienna, Austria.
[4]Department of Dermatology, Research Division of Biology and Pathobiology of the Skin, Medical University Vienna, Austria
[5] Christian Doppler Laboratory for Laboratory Biotechnology of Skin Aging, Medical, Austria
[6]Centre of Physiology and Pharmacology, Institute of Physiology, Medical University of Vienna, Austria
[7]Department of Cardiology, Medical University Vienna, Vienna, Austria
[8]Molecular Neuro-Oncology Research Unit, Department of Pediatrics and Adolescent Medicine and Institute of Neurology, Medical University of Vienna, Austria
[9]Red Cross Blood Transfusion Service of Upper Austria, Linz, Austria

[*]These authors share the last and corresponding authorship.

Corresponding authors:

Hendrik Jan Ankersmit, MD
Department of Thoracic Surgery, Medical University of Vienna
Christian Doppler Laboratory for Cardiac and Thoracic Diagnosis and Regeneration
Währinger Gürtel 18-20
1090 Vienna, Austria
Tel.: +43-1-40400-67770
Fax.: +43-1-40400-69770
e-mail: hendrik.ankersmit@meduniwien.ac.at

Michael Mildner, PhD
Medical University of Vienna
Department of Dermatology,
Research Division of Biology and Pathobiology of the Skin,
Lazarettgasse14
1090 Vienna, Austria
Tel: +43-1-40400-73507
Fax: +43-1-40400-73590
e-mail: michael.mildner@meduniwien.ac.at

Supplementary Figure S1

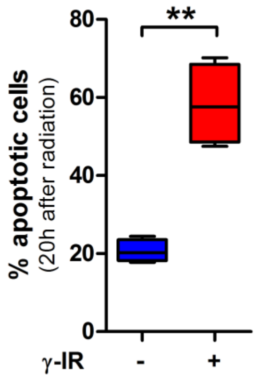

Supplementary Figure S1

Ionizing irradiation induces apoptosis in human PBMCs. The number of annexin V-fluorescein/propidium iodide (FITC/PI) positive PBMCs was measured at 20h after cultivation. **$p < 0.01$; $n = 4$.

Supplementary Figure S2

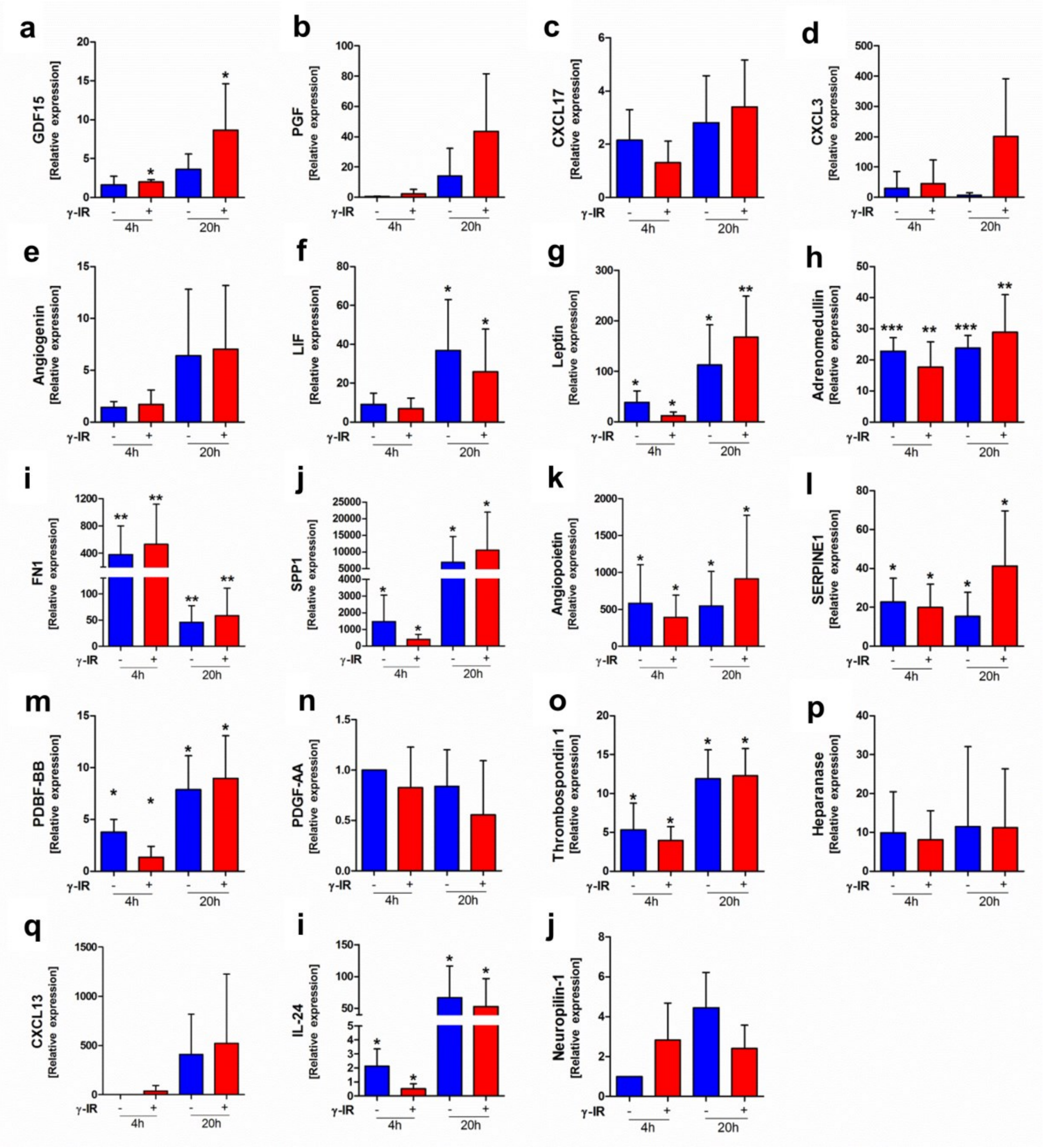

Supplementary Figure S2

Quantitative PCR results show expression of genes involved in angiogenesis, wound healing, or chemotaxis. (a-s) In comparing levels at 4 h and 20 h, most genes showed a time dependent increase, except FN1 (i) and PDGF-AA (n). A few genes tended to show higher expression in irradiated (+, red) compared to non-irradiated (-, blue) PBMCs. Expression values were normalized to the gene expression values at 0 h. B2M served as an internal control gene. Data represent the mean ± SD of four samples; *p<0.05; **p<0.01; ***p<0.001

Supplementary Figure S3

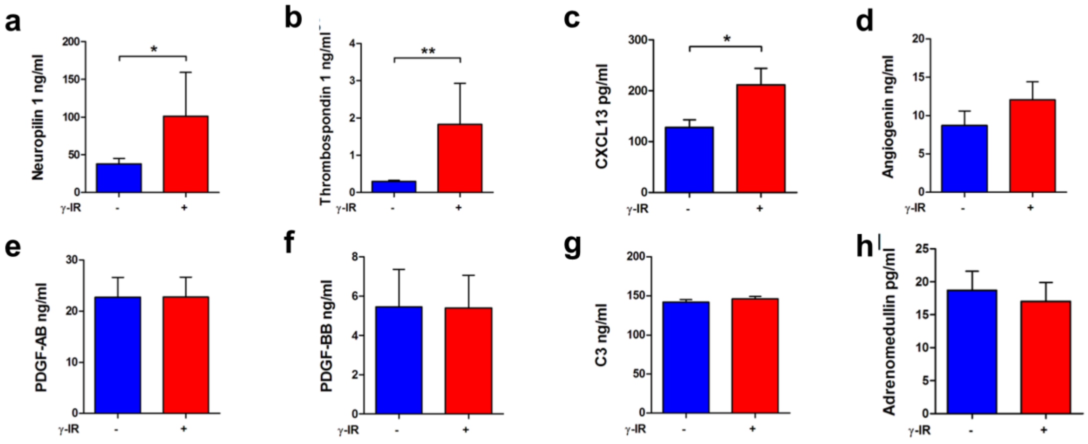

Supplementary Figure S3

ELISA detection identified proteins isolated from the CM of non-irradiated and irradiated PBMCs measured after 20 h of culture. Cell culture medium alone (without cells) did not contain detectable amounts of any measured mediators. Protein concentrations of (a) neuropilin, (b) thrombospondin, and (c) CXCL13 were significantly higher in CM from irradiated (+, red) than in non-irradiated (-, blue) PBMCs; however, levels of (d) angiogenin, (e) PDGF-AB, (f) PDGF-BB, (g) C3, and (h) adrenomedullin were comparable in both groups. Data are given as mean ± SD of five samples; *p<0.05; **p<0.01.

Supplementary Figure S4

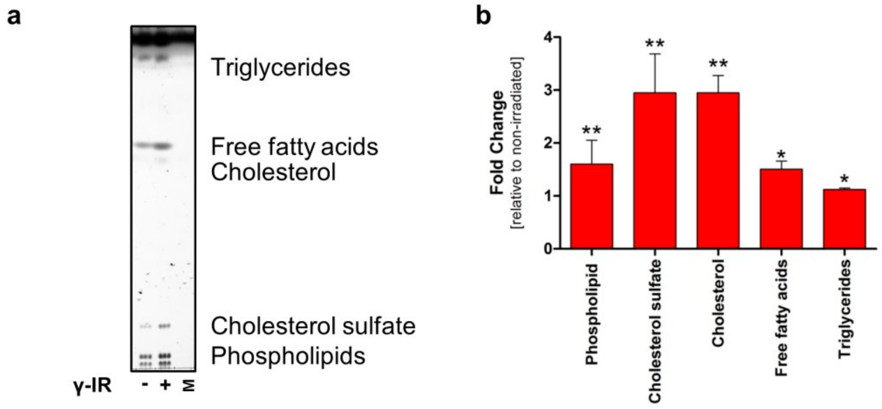

Supplementary Figure S4

Ionizing radiation induced the release of soluble and membrane-bound lipids from human PBMCs. PBMCs were either irradiated (+) or non-irradiated (-), and cultured for 24 h. Then, the cells and cell debris were removed by serial centrifugation, and the lipids were isolated with a chloroform/methanol extraction protocol. (a) Dissolved lipids were resolved with thin layer chromatography (TLC; Pappinen et al.). Cell medium (M) cultured without cells did not contain any lipids. The CM of PBMCs contained triglycerides, free fatty acids, cholesterol, cholesterol sulfate, and phospholipids. (b) Quantitative analysis of TLC data showed that the CM samples from irradiated PBMCs had significant increases in absolute concentrations of all lipid classes compared to non-irradiated samples. Data are expressed as the mean ± SD of four samples; *p<0.05; **p<0.01.

Supplementary Figure S5

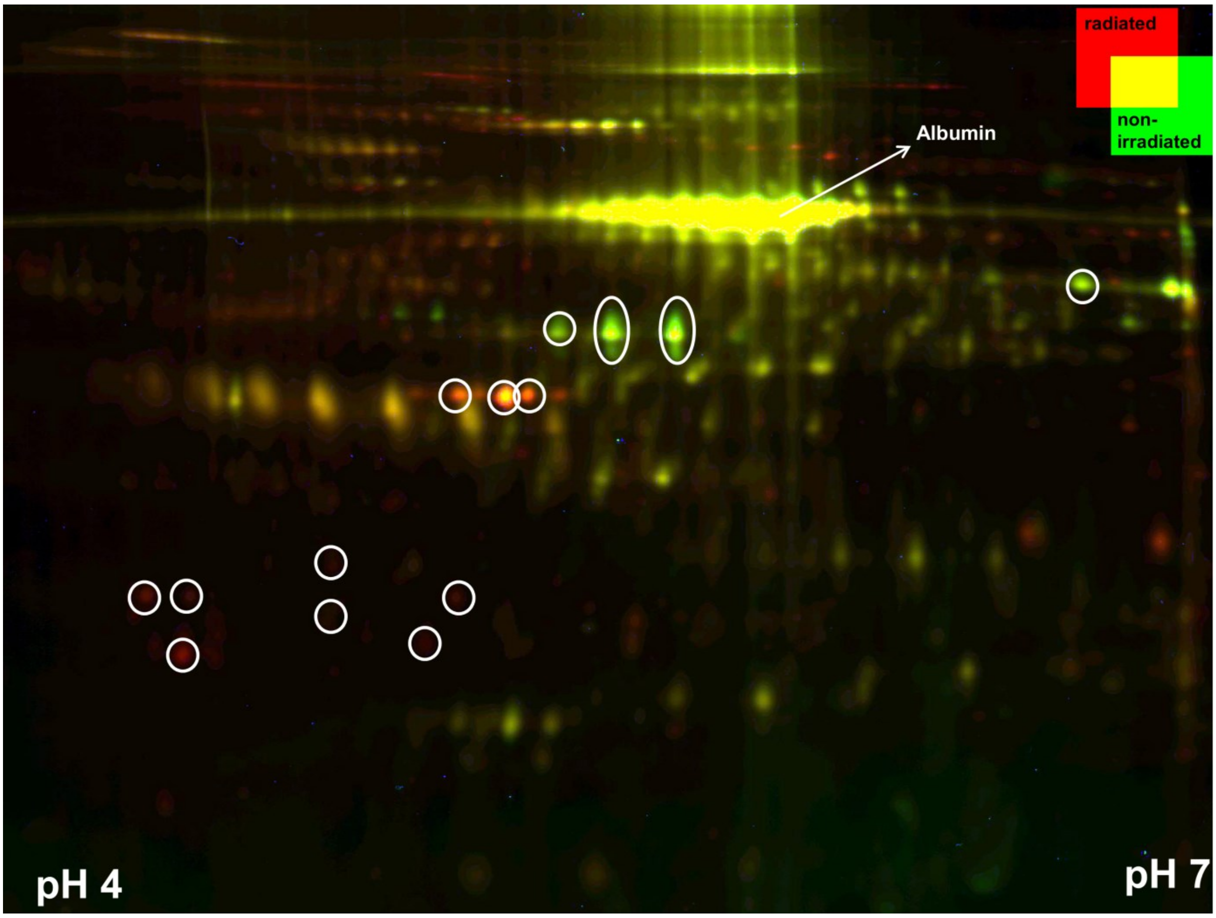

Supplementary Figure S5

2D-Difference gel electrophoresis results show different exosome protein contents of non-irradiated and irradiated PBMCs. The total CyDye-labeled, exosome protein extract sample (45 µg) comprised a combination of four samples (15 µg each pooled from 4 donors): The proteins were separated in the pH range of 4-7. The red and green spots are proteins that showed increased and decreased expression, respectively, in exosomes from irradiated PBMCs, compared to exosomes from non-irradiated PBMCs. Yellow spots are proteins that show comparable concentrations in exosomes from irradiated and non-irradiated PBMCs. The white circles indicate proteins that were detected in irradiated or non-irradiated samples exclusively. The large protein spot at 66 kDa represents the albumin fraction (arrow). Exosomes from 4 donors were used for this experiment.

Supplementary Figure S6

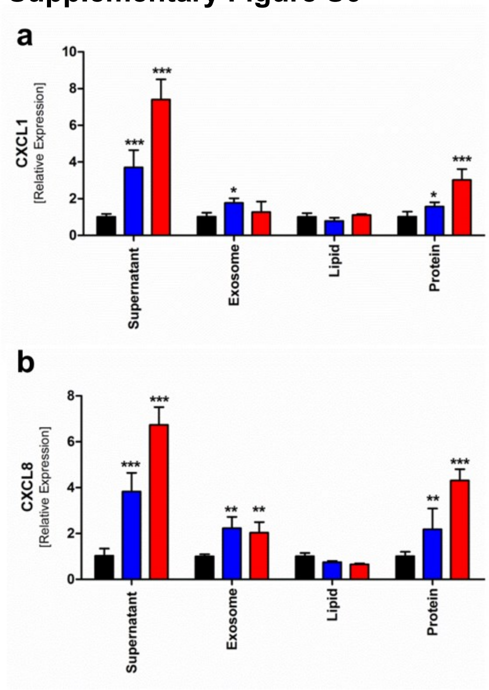

Supplementary Figure S6

Exosomes and proteins stimulate CXCL1 and CLXCL8 expression in keratinocytes. Keratinocyte gene expression of (a) CXCL1 and (b) CXCL8 was measured relative to B2M expression (control). Keratinocytes were unstimulated (black bars) or stimulated with total CM (supernatant) or the indicated CM fractions, collected from irradiated (red bars) or non-irradiated (blue bars) PBMCs. RNA was isolated 6 h after cell stimulation. Bars represent the mean ± SD of n=2 experiments performed in triplicate.*p<0.05; **p<0.01; ***p<0.001.

Supplementary Figure S7

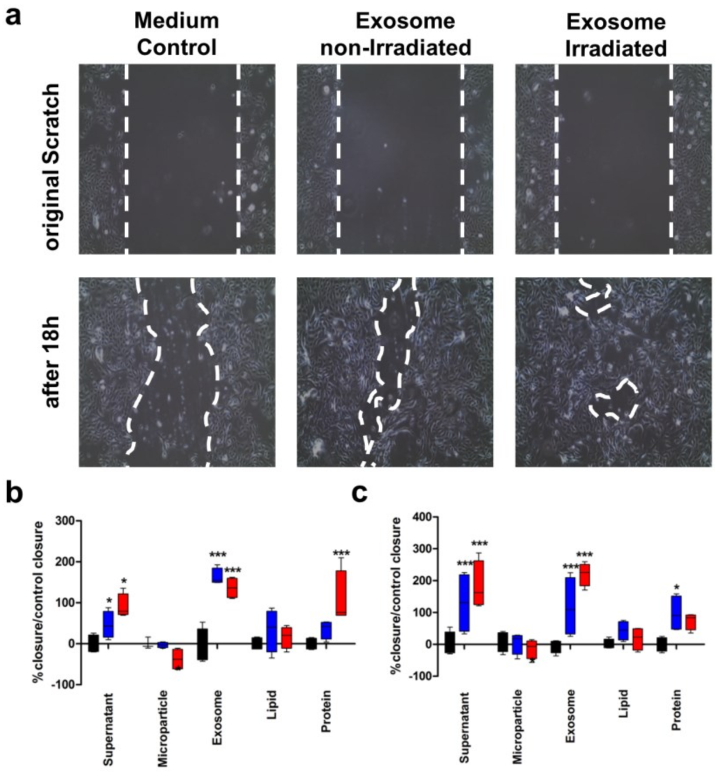

Supplementary Figure S7

Exosomes and CM proteins enhance keratinocyte migration. (A, *top*) Keratinocyte monolayers were scratched (clear areas outlined in white dashed lines) to simulate a wound. Wounds were untreated or treated with exosome preparations from non-irradiated or irradiated PBMCs. (*Bottom*) After 18 h, fibroblast proliferation began to close the wound in (*left*) untreated and (*middle*, *right*) treated cultures. Treatment with PBMC-derived exosomes accelerated wound closure. 10 fold magnification (b) Wound areas were measured in 8 scratches after 24 h. The percentage of closure compared to closure in control (untreated) was calculated. (c) The same experiment was performed with keratinocytes, but without using 10% CellGro medium as basal medium. In this experimental setup, the proliferative effects of primary CM, exosomes, and CM proteins were more pronounced than the effects observed in the initial experimental setting. Data are expressed as the mean ± SD of two independent experiments. *p<0.05; ***p<0.001.

Supplementary Figure S8

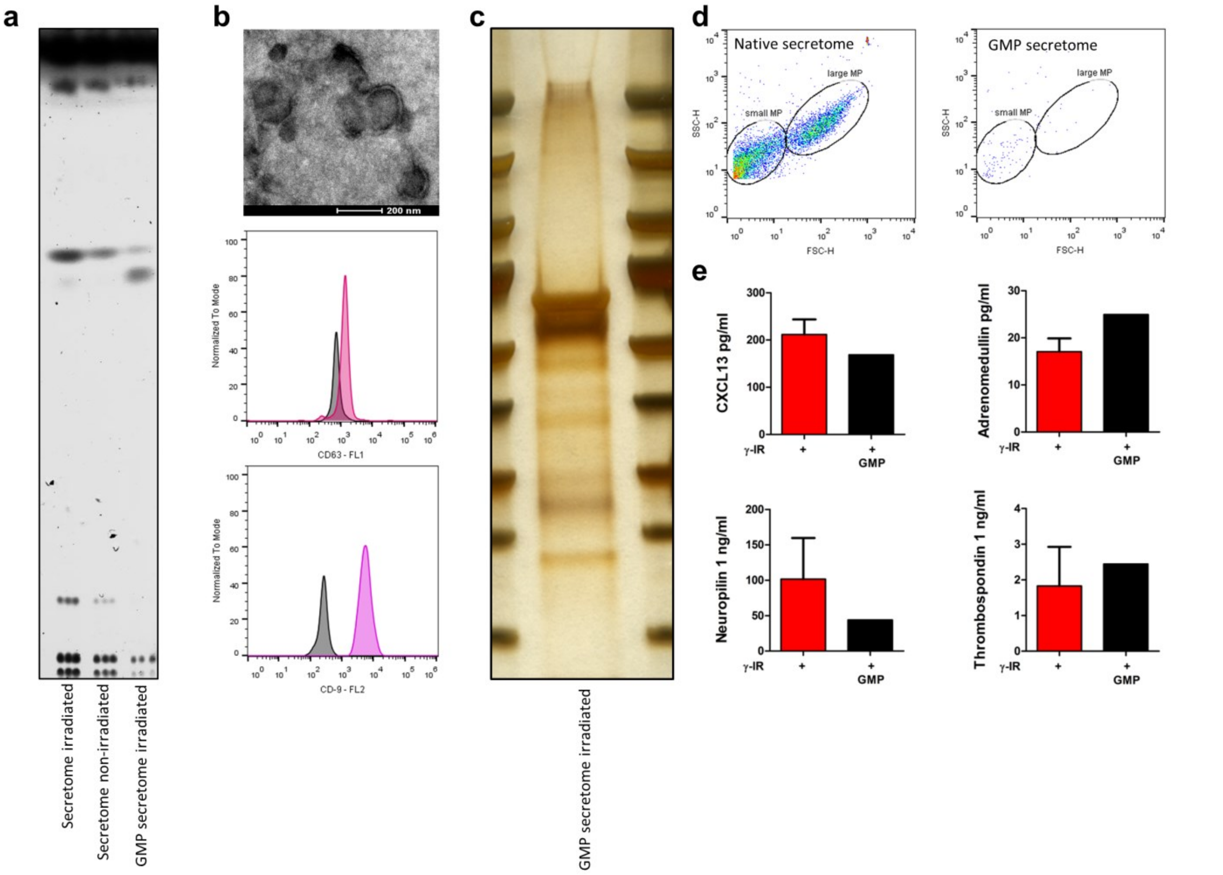

Supplementary Figure S8

Comparability of biological components of the PBMC secretome, isolated with experimental methods or under GMP conditions. (a) TLC shows secretomes from CM isolated from irradiated (*line 1*) and non-irradiated (*line 2*) PBMCs, and CM from irradiated PBMCs, but processed under GMP-compliant conditions (*line 3*). The absolute concentration of lipids was lower in the GMP-compliant CM; however, except for cholesterol, the lipid compositions were comparable in the two different preparations. (b) *Top:* TEM image of exosomes derived from GMP-compliant CM showed vesicles with a diameter of ~100 nm. Scale bar = 200 nm. (*Middle, bottom*) FACS analysis showed that these vesicles were positive for CD63 and CD9, as indicated. (c) After exosomes were lysed, the proteins were separated with 1-D gel electrophoresis and stained with silver stain. Several different protein bands were detected with quality comparable to that observed in exosomes derived from CM with the experimental protocols. (d) GMP-compliant CM did not contain considerable numbers of microparticles. In

the GMP protocol, microparticles were removed by filtration through 0.2-μm filters. (e) Selected CM proteins were compared between experimental (+, red) and GMP-compliant (black) preparations. Similar concentrations were detectable for CXCL13, andrenomedullin, and thrombospondin. Neuropilin concentrations tended to be lower in GMP-compliant CM.

Supplementary Table 1: Enrichment of GO-terms in up-regulated genes coding for secretory proteins in **irradiated** PBMCs.

GO-Term	Enriched terms	p-value	Enrichment Score	Number of Genes
GO:0001568	blood vessel development	1.53e-07	5.32	22
GO:0045766	positive regulation of angiogenesis	3.00e-05	6.34	10
GO:0042060	wound healing	0.0012	2.74	21
GO:0032502	developmental processes	0.0007	1.65	83
GO:0007599	hemostasis	0.0007	3.08	19
GO:0050819	negative regulation of coagulation	0.0007	11.68	6
GO:0050900	leukocyte migration	0.0007	4.28	13

Biological Function of the 213 gene products. The 213 genes were classified into different biological processes. Biological processes that were over-represented in the data set relative to the frequency of genes in a reference database with a corrected p value <0.05 are given.

Enrichment of GO-terms in up-regulated genes coding for secretory proteins in **non-irradiated** PBMCs.

GO-Term	Enriched terms	p-value	Enrichment Score	Number of Genes
GO:0051955	regulation of amino acid transport	0.0403	15.39	3
GO:0050855	regulation of B cell receptor signaling pathway	0.0490	33.71	2
GO:0044060	regulation of endocrine process	0.0116	20.52	4

Biological Function of the 179 gene products. The 179 genes were classified into different biological processes. Biological processes that were over-represented in the data set relative to the frequency of genes in a reference database with a corrected p value <0.05 are given.

Supplementary Table 2: 213 Up-regulated transcripts in **irradiated** PBMC coding for secretory proteins

Gene Symbol	EntrezGene	Gene Name
C16orf74	404550	chromosome 16 open reading frame 74
CYP17A1	1586	cytochrome P450, family 17, subfamily A, polypeptide 1
HIST1H2AD	3013	histone cluster 1, H2ad
TESK2	10420	testis-specific kinase 2
GSTT2	2953	glutathione S-transferase theta 2
P4HA1	5033	prolyl 4-hydroxylase, alpha polypeptide I
SPINK1	6690	serine peptidase inhibitor, Kazal type 1
RRAD	6236	Ras-related associated with diabetes
TIPARP	25976	TCDD-inducible poly(ADP-ribose) polymerase
HIST1H2AK	8330	histone cluster 1, H2ak
SERPINE1	5054	serpin peptidase inhibitor, clade E (nexin, plasminogen activator inhibitor type 1), member 1
CHMP1B	57132	charged multivesicular body protein 1B
VCAN	1462	versican
AHRR	57491	aryl-hydrocarbon receptor repressor
UBE2A	7319	ubiquitin-conjugating enzyme E2A
IBA57	200205	IBA57, iron-sulfur cluster assembly homolog (S. cerevisiae)
MDGA1	266727	MAM domain containing glycosylphosphatidylinositol anchor 1
MANF	7873	mesencephalic astrocyte-derived neurotrophic factor
IVNS1ABP	10625	influenza virus NS1A binding protein
CXCL3	2921	chemokine (C-X-C motif) ligand 3
EYA3	2140	eyes absent homolog 3 (Drosophila)
SH2D6	284948	SH2 domain containing 6
EDARADD	128178	EDAR-associated death domain
TGIF1	7050	TGFB-induced factor homeobox 1
LRRC6	23639	leucine rich repeat containing 6
ALCAM	214	activated leukocyte cell adhesion molecule
MSL1	339287	male-specific lethal 1 homolog (Drosophila)
IL24	11009	interleukin 24
ASPHD1	253982	aspartate beta-hydroxylase domain containing 1
GDF15	9518	growth differentiation factor 15
GRB10	2887	growth factor receptor-bound protein 10
LPO	4025	lactoperoxidase
STX5	6811	syntaxin 5
PLEKHH3	79990	pleckstrin homology domain containing, family H (with MyTH4 domain) member 3
RAB42	115273	RAB42, member RAS oncogene family
FANCL	55120	Fanconi anemia, complementation group L
P4HA2	8974	prolyl 4-hydroxylase, alpha polypeptide II
S100A5	6276	S100 calcium binding protein A5
MFAP4	4239	microfibrillar-associated protein 4
GATSL3	652968	GATS protein-like 3
PATE2	399967	prostate and testis expressed 2
FLT1	2321	fms-related tyrosine kinase 1 (vascular endothelial growth factor/vascular permeability factor receptor)
DENND5A	23258	DENN/MADD domain containing 5A
DLG4	1742	discs, large homolog 4 (Drosophila)
DRGX	644168	dorsal root ganglia homeobox
LAD1	3898	ladinin 1
CST6	1474	cystatin E/M

GNA12	2768	guanine nucleotide binding protein (G protein) alpha 12
IL23A	51561	interleukin 23, alpha subunit p19
SEC24A	10802	SEC24 family, member A (S. cerevisiae)
TSHB	7252	thyroid stimulating hormone, beta
PPIF	10105	peptidylprolyl isomerase F
TBX6	6911	T-box 6
KCNE1	3753	potassium voltage-gated channel, Isk-related family, member 1
IL1A	3552	interleukin 1, alpha
GADD45G	10912	growth arrest and DNA-damage-inducible, gamma
FHL3	2275	four and a half LIM domains 3
NAGS	162417	N-acetylglutamate synthase
ACOX2	8309	acyl-CoA oxidase 2, branched chain
EFEMP2	30008	EGF containing fibulin-like extracellular matrix protein 2
AK2	204	adenylate kinase 2
PDE2A	5138	phosphodiesterase 2A, cGMP-stimulated
TUBB2A	7280	tubulin, beta 2A class IIa
VWCE	220001	von Willebrand factor C and EGF domains
TFPI2	7980	tissue factor pathway inhibitor 2
TOB2	10766	transducer of ERBB2, 2
THBS1	7057	thrombospondin 1
MFI2	4241	antigen p97 (melanoma associated) identified by monoclonal antibodies 133.2 and 96.5
SMOX	54498	spermine oxidase
IFNA7	3444	interferon, alpha 7
CDKN1A	1026	cyclin-dependent kinase inhibitor 1A (p21, Cip1)
BBC3	27113	BCL2 binding component 3
PCOLCE2	26577	procollagen C-endopeptidase enhancer 2
PPFIA1	8500	protein tyrosine phosphatase, receptor type, f polypeptide (PTPRF), interacting protein (liprin), alpha 1
NOTCH3	4854	notch 3
PLD2	5338	phospholipase D2
PPP1R3B	79660	protein phosphatase 1, regulatory subunit 3B
H2AFX	3014	H2A histone family, member X
ALOX15B	247	arachidonate 15-lipoxygenase, type B
HIST1H2AG	8969	histone cluster 1, H2ag
CXCL13	10563	chemokine (C-X-C motif) ligand 13
PDGFB	5155	platelet-derived growth factor beta polypeptide
RNASET2	8635	ribonuclease T2
NRP1	8829	neuropilin 1
LIF	3976	leukemia inhibitory factor
LACC1	144811	laccase (multicopper oxidoreductase) domain containing 1
TIMP1	7076	TIMP metallopeptidase inhibitor 1
PRSS53	339105	protease, serine, 53
PINLYP	390940	phospholipase A2 inhibitor and LY6/PLAUR domain containing
ANGPTL4	51129	angiopoietin-like 4
LEP	3952	leptin
ERO1L	30001	ERO1-like (S. cerevisiae)
RGL3	57139	ral guanine nucleotide dissociation stimulator-like 3
PPP1R3G	648791	protein phosphatase 1, regulatory subunit 3G
CXCL5	6374	chemokine (C-X-C motif) ligand 5
COL27A1	85301	collagen, type XXVII, alpha 1
PLOD2	5352	procollagen-lysine, 2-oxoglutarate 5-dioxygenase 2
C4orf47	441054	chromosome 4 open reading frame 47
NMNAT1	64802	nicotinamide nucleotide adenylyltransferase 1
DCAF4L2	138009	DDB1 and CUL4 associated factor 4-like 2

IGLON5	402665	IgLON family member 5
FUT11	170384	fucosyltransferase 11 (alpha (1,3) fucosyltransferase)
C16orf72	29035	chromosome 16 open reading frame 72
PELI3	246330	pellino E3 ubiquitin protein ligase family member 3
EPPIN-WFDC6	100526773	EPPIN-WFDC6 readthrough
RGS13	6003	regulator of G-protein signaling 13
RSPH9	221421	radial spoke head 9 homolog (Chlamydomonas)
GPNMB	10457	glycoprotein (transmembrane) nmb
PCNA	5111	proliferating cell nuclear antigen
RND1	27289	Rho family GTPase 1
TMEM189	387521	transmembrane protein 189
C20orf96	140680	chromosome 20 open reading frame 96
AVPI1	60370	arginine vasopressin-induced 1
THAP8	199745	THAP domain containing 8
LACTB	114294	lactamase, beta
FGF11	2256	fibroblast growth factor 11
ATP7A	538	ATPase, Cu++ transporting, alpha polypeptide
NIM1	167359	serine/threonine-protein kinase NIM1
INHBA	3624	inhibin, beta A
HIST1H2AI	8329	histone cluster 1, H2ai
RBP4	5950	retinol binding protein 4, plasma
C6orf170	221322	chromosome 6 open reading frame 170
HIST1H2AC	8334	histone cluster 1, H2ac
TGM2	7052	transglutaminase 2 (C polypeptide, protein-glutamine-gamma-glutamyltransferase)
QSOX1	5768	quiescin Q6 sulfhydryl oxidase 1
TKTL1	8277	transketolase-like 1
SERTAD1	29950	SERTA domain containing 1
PDLIM7	9260	PDZ and LIM domain 7 (enigma)
NPTX1	4884	neuronal pentraxin I
AGRN	375790	agrin
COL7A1	1294	collagen, type VII, alpha 1
WBP5	51186	WW domain binding protein 5
CIRBP	1153	cold inducible RNA binding protein
SLC5A6	8884	solute carrier family 5 (sodium-dependent vitamin transporter), member 6
HIST1H2AL	8332	histone cluster 1, H2al
ADM	133	adrenomedullin
IGF2	3481	insulin-like growth factor 2 (somatomedin A)
PGF	5228	placental growth factor
PDK4	5166	pyruvate dehydrogenase kinase, isozyme 4
PLA2G7	7941	phospholipase A2, group VII (platelet-activating factor acetylhydrolase, plasma)
SPP1	6696	secreted phosphoprotein 1
ZNF697	90874	zinc finger protein 697
HIST1H2AM	8336	histone cluster 1, H2am
TP53INP2	58476	tumor protein p53 inducible nuclear protein 2
S100A11	6282	S100 calcium binding protein A11
HTRA1	5654	HtrA serine peptidase 1
DFNA5	1687	deafness, autosomal dominant 5
EFNA3	1944	ephrin-A3
FOXR1	283150	forkhead box R1
INPP1	3628	inositol polyphosphate-1-phosphatase
SLC25A19	60386	solute carrier family 25 (mitochondrial thiamine pyrophosphate carrier), member 19

ADAMDEC1	27299	ADAM-like, decysin 1
EGLN3	112399	egl nine homolog 3 (C. elegans)
ENO2	2026	enolase 2 (gamma, neuronal)
RAB17	64284	RAB17, member RAS oncogene family
ARL8B	55207	ADP-ribosylation factor-like 8B
GPX3	2878	glutathione peroxidase 3 (plasma)
MATN1	4146	matrilin 1, cartilage matrix protein
ADAMTSL4	54507	ADAMTS-like 4
WDR54	84058	WD repeat domain 54
ANXA5	308	annexin A5
FGF5	2250	fibroblast growth factor 5
PLAT	5327	plasminogen activator, tissue
LYPD3	27076	LY6/PLAUR domain containing 3
METTL12	751071	methyltransferase like 12
VMO1	284013	vitelline membrane outer layer 1 homolog (chicken)
ITGBL1	9358	integrin, beta-like 1 (with EGF-like repeat domains)
GATA4	2626	GATA binding protein 4
SERAC1	84947	serine active site containing 1
GNA15	2769	guanine nucleotide binding protein (G protein), alpha 15 (Gq class)
COL16A1	1307	collagen, type XVI, alpha 1
SIX3	6496	SIX homeobox 3
IER5L	389792	immediate early response 5-like
MICALL2	79778	MICAL-like 2
ZNF808	388558	zinc finger protein 808
CD109	135228	CD109 molecule
SFTPA1	653509	surfactant protein A1
IL18BP	10068	interleukin 18 binding protein
GSTT2B	653689	glutathione S-transferase theta 2B (gene/pseudogene)
FCAR	2204	Fc fragment of IgA, receptor for
RAB13	5872	RAB13, member RAS oncogene family
FN1	2335	fibronectin 1
CLLU1OS	574016	chronic lymphocytic leukemia up-regulated 1 opposite strand
DHRS9	10170	dehydrogenase/reductase (SDR family) member 9
GADD45B	4616	growth arrest and DNA-damage-inducible, beta
PLIN5	440503	perilipin 5
SLC26A6	65010	solute carrier family 26, member 6
EIF4A3	9775	eukaryotic translation initiation factor 4A3
DPCD	25911	deleted in primary ciliary dyskinesia homolog (mouse)
RFX2	5990	regulatory factor X, 2 (influences HLA class II expression)
PRSS37	136242	protease, serine, 37
SLAMF9	89886	SLAM family member 9
HPSE	10855	heparanase
UBE2V1	7335	ubiquitin-conjugating enzyme E2 variant 1
EPPIN	57119	epididymal peptidase inhibitor
TPI1	7167	triosephosphate isomerase 1
PROC	5624	protein C (inactivator of coagulation factors Va and VIIIa)
EGFL7	51162	EGF-like-domain, multiple 7
DEFB128	245939	defensin, beta 128
PLXNB3	5365	plexin B3
ATF5	22809	activating transcription factor 5
ANGPT4	51378	angiopoietin 4
TGM5	9333	transglutaminase 5
ANG1	7556	Angiogenin
MOB3B	79817	MOB kinase activator 3B

MMP9	4318	matrix metallopeptidase 9 (gelatinase B, 92kDa gelatinase, 92kDa type IV collagenase)
WDR74	54663	WD repeat domain 74
TFPI	7035	tissue factor pathway inhibitor (lipoprotein-associated coagulation inhibitor)
ST8SIA4	7903	ST8 alpha-N-acetyl-neuraminide alpha-2,8-sialyltransferase 4
SCUBE1	80274	signal peptide, CUB domain, EGF-like 1
C3	718	complement component 3
NOL3	8996	nucleolar protein 3 (apoptosis repressor with CARD domain)
PLXNA3	55558	plexin A3

The list was generated by positive selection of up-regulated genes in irradiated PBMC with SignalP and SecretomeP analysis followed by negative selection with TMHMM analyisis excluding proteins with transmembrane helices.

Supplementary Table 3: 179 Up-regulated transcripts in **non-irradiated** PBMC coding for secretory proteins

Gene Symbol	EntrezGene	Gene Name
HIST1H2AD	3013	histone cluster 1, H2ad
C1orf151-NBL1	100532736	C1orf151-NBL1 readthrough
IDS	3423	iduronate 2-sulfatase
UFSP2	55325	UFM1-specific peptidase 2
ZNF143	7702	zinc finger protein 143
TUBE1	51175	tubulin, epsilon 1
NXPH4	11247	neurexophilin 4
AGXT2L2	85007	alanine-glyoxylate aminotransferase 2-like 2
P4HA1	5033	prolyl 4-hydroxylase, alpha polypeptide I
TTC9C	283237	tetratricopeptide repeat domain 9C
SUPV3L1	6832	suppressor of var1, 3-like 1 (S. cerevisiae)
SDS	10993	serine dehydratase
VPS37A	137492	vacuolar protein sorting 37 homolog A (S. cerevisiae)
HIST1H2AK	8330	histone cluster 1, H2ak
VASH2	79805	vasohibin 2
PPM1K	152926	protein phosphatase, Mg2+/Mn2+ dependent, 1K
RSPRY1	89970	ring finger and SPRY domain containing 1
PHRF1	57661	PHD and ring finger domains 1
AHRR	57491	aryl-hydrocarbon receptor repressor
EPAS1	2034	endothelial PAS domain protein 1
XPA	7507	xeroderma pigmentosum, complementation group A
IVNS1ABP	10625	influenza virus NS1A binding protein
SPOCK2	9806	sparc/osteonectin, cwcv and kazal-like domains proteoglycan (testican) 2
IL4R	3566	interleukin 4 receptor
ZNF250	58500	zinc finger protein 250
ALCAM	214	activated leukocyte cell adhesion molecule
N4BP2L1	90634	NEDD4 binding protein 2-like 1
IL24	11009	interleukin 24
ZNF563	147837	zinc finger protein 563
WDFY2	115825	WD repeat and FYVE domain containing 2
KRTAP4-8	728224	keratin associated protein 4-8
MAP1LC3A	84557	microtubule-associated protein 1 light chain 3 alpha
B3GALNT2	148789	beta-1,3-N-acetylgalactosaminyltransferase 2
LPO	4025	lactoperoxidase
RPL28	6158	ribosomal protein L28
SRSF1	6426	serine/arginine-rich splicing factor 1
C15orf53	400359	chromosome 15 open reading frame 53
P4HA2	8974	prolyl 4-hydroxylase, alpha polypeptide II
NUP43	348995	nucleoporin 43kDa
CHORDC1	26973	cysteine and histidine-rich domain (CHORD) containing 1
SNED1	25992	sushi, nidogen and EGF-like domains 1
SP140	11262	SP140 nuclear body protein
VENTX	27287	VENT homeobox
FEZ1	9638	fasciculation and elongation protein zeta 1 (zygin I)
EBLN2	55096	endogenous Bornavirus-like nucleoprotein 2
FAM115C	285966	family with sequence similarity 115, member C
PDK1	5163	pyruvate dehydrogenase kinase, isozyme 1
ACAN	176	aggrecan
APOL4	80832	apolipoprotein L, 4

RELB	5971	v-rel reticuloendotheliosis viral oncogene homolog B
RCOR1	23186	REST corepressor 1
ETV6	2120	ets variant 6
ACTRT3	84517	actin-related protein T3
PPP1R16B	26051	protein phosphatase 1, regulatory subunit 16B
KIF3A	11127	kinesin family member 3A
SEC24A	10802	SEC24 family, member A (S. cerevisiae)
DOCK9	23348	dedicator of cytokinesis 9
ZBTB25	7597	zinc finger and BTB domain containing 25
TBX6	6911	T-box 6
TCP1	6950	t-complex 1
NBL1	4681	neuroblastoma, suppression of tumorigenicity 1
AK2	204	adenylate kinase 2
THAP6	152815	THAP domain containing 6
FST	10468	follistatin
YPEL4	219539	yippee-like 4 (Drosophila)
YPEL5	51646	yippee-like 5 (Drosophila)
SERPINH1	871	serpin peptidase inhibitor, clade H (heat shock protein 47), member 1, (collagen binding protein 1)
ARPP19	10776	cAMP-regulated phosphoprotein, 19kDa
ZNF230	7773	zinc finger protein 230
PHF20L1	51105	PHD finger protein 20-like 1
SMAD7	4092	SMAD family member 7
RNF145	153830	ring finger protein 145
GK	2710	glycerol kinase
HIST1H2AG	8969	histone cluster 1, H2ag
CMSS1	84319	cms1 ribosomal small subunit homolog (yeast)
MTMR1	8776	myotubularin related protein 1
RNASET2	8635	ribonuclease T2
CHD1	1105	chromodomain helicase DNA binding protein 1
HSPB11	51668	heat shock protein family B (small), member 11
CACYBP	27101	calcyclin binding protein
ERO1L	30001	ERO1-like (S. cerevisiae)
LEP	3952	leptin
RAB8B	51762	RAB8B, member RAS oncogene family
KLHL28	54813	kelch-like 28 (Drosophila)
SDCBP2	27111	syndecan binding protein (syntenin) 2
ZFP42	132625	zinc finger protein 42 homolog (mouse)
C4orf47	441054	chromosome 4 open reading frame 47
FUT11	170384	fucosyltransferase 11 (alpha (1,3) fucosyltransferase)
CHM	1121	choroideremia (Rab escort protein 1)
CHKA	1119	choline kinase alpha
DNAJB4	11080	DnaJ (Hsp40) homolog, subfamily B, member 4
GPNMB	10457	glycoprotein (transmembrane) nmb
C1orf74	148304	chromosome 1 open reading frame 74
NDRG4	65009	NDRG family member 4
LRP5L	91355	low density lipoprotein receptor-related protein 5-like
BAGE	574	B melanoma antigen
KRAS	3845	v-Ki-ras2 Kirsten rat sarcoma viral oncogene homolog
JHDM1D	80853	jumonji C domain containing histone demethylase 1 homolog D (S. cerevisiae)
ATP7A	538	ATPase, Cu++ transporting, alpha polypeptide
HIST1H2AI	8329	histone cluster 1, H2ai
INHBA	3624	inhibin, beta A
CSH1	1442	chorionic somatomammotropin hormone 1 (placental lactogen)
HIST1H2AC	8334	histone cluster 1, H2ac

KIAA1147	57189	KIAA1147
TKTL1	8277	transketolase-like 1
COL7A1	1294	collagen, type VII, alpha 1
PHLDB3	653583	pleckstrin homology-like domain, family B, member 3
RAB33A	9363	RAB33A, member RAS oncogene family
WBP5	51186	WW domain binding protein 5
JAG1	182	jagged 1
HIST1H2AL	8332	histone cluster 1, H2al
ADAMTS17	170691	ADAM metallopeptidase with thrombospondin type 1 motif, 17
ZNF616	90317	zinc finger protein 616
TRH	7200	thyrotropin-releasing hormone
SPP1	6696	secreted phosphoprotein 1
PASK	23178	PAS domain containing serine/threonine kinase
TSC1	7248	tuberous sclerosis 1
SENP2	59343	SUMO1/sentrin/SMT3 specific peptidase 2
ZNF562	54811	zinc finger protein 562
LPXN	9404	leupaxin
CBFA2T2	9139	core-binding factor, runt domain, alpha subunit 2; translocated to, 2
HIST1H2AM	8336	histone cluster 1, H2am
SERPINA1	5265	serpin peptidase inhibitor, clade A (alpha-1 antiproteinase, antitrypsin), member 1
GMNN	51053	geminin, DNA replication inhibitor
CTSL1	1514	cathepsin L1
CNOT2	4848	CCR4-NOT transcription complex, subunit 2
POLR2M	81488	polymerase (RNA) II (DNA directed) polypeptide M
ENO2	2026	enolase 2 (gamma, neuronal)
RAB17	64284	RAB17, member RAS oncogene family
SAR1B	51128	SAR1 homolog B (S. cerevisiae)
OXSM	54995	3-oxoacyl-ACP synthase, mitochondrial
CSH2	1443	chorionic somatomammotropin hormone 2
AK4	205	adenylate kinase 4
GFPT2	9945	glutamine-fructose-6-phosphate transaminase 2
FAM131A	131408	family with sequence similarity 131, member A
CWC25	54883	CWC25 spliceosome-associated protein homolog (S. cerevisiae)
LOH12CR1	118426	loss of heterozygosity, 12, chromosomal region 1
C1orf21	81563	chromosome 1 open reading frame 21
CAMK2N2	94032	calcium/calmodulin-dependent protein kinase II inhibitor 2
CD109	135228	CD109 molecule
NDRG1	10397	N-myc downstream regulated 1
RGCC	28984	regulator of cell cycle
GFI1	2672	growth factor independent 1 transcription repressor
ZCCHC18	644353	zinc finger, CCHC domain containing 18
KDM2B	84678	lysine (K)-specific demethylase 2B
CCDC47	57003	coiled-coil domain containing 47
UBR2	23304	ubiquitin protein ligase E3 component n-recognin 2
PLIN5	440503	perilipin 5
RAPH1	65059	Ras association (RalGDS/AF-6) and pleckstrin homology domains 1
CCNH	902	cyclin H
NSUN4	387338	NOP2/Sun domain family, member 4
HSP90AA1	3320	heat shock protein 90kDa alpha (cytosolic), class A member 1
AP3M2	10947	adaptor-related protein complex 3, mu 2 subunit
LOC100507855	100507855	adenylate kinase isoenzyme 4, mitochondrial-like
NAA30	122830	N(alpha)-acetyltransferase 30, NatC catalytic subunit
CYSTM1	84418	cysteine-rich transmembrane module containing 1
DNAJA4	55466	DnaJ (Hsp40) homolog, subfamily A, member 4
RSC1A1	6248	regulatory solute carrier protein, family 1, member 1

ST6GAL1	6480	ST6 beta-galactosamide alpha-2,6-sialyltranferase 1
CHTOP	26097	chromatin target of PRMT1
NR3C1	2908	nuclear receptor subfamily 3, group C, member 1 (glucocorticoid receptor)
PTPRC	5788	protein tyrosine phosphatase, receptor type, C
PLXNA3	55558	plexin A3
ANKRD37	353322	ankyrin repeat domain 37
CCNDBP1	23582	cyclin D-type binding-protein 1

The list was generated by positive selection of up-regulated genes in non-irradiated PBMC with SignalP and SecretomeP analysis followed by negative selection with TMHMM analyisis excluding proteins with transmembrane helices.
